# Association Between the cMIND Diet Pattern, Psychological Resilience and Cognitive Decline Trajectories: Findings from a Prospective Cohort Study in China

**DOI:** 10.3390/nu18213608

**Published:** 2026-10-30

**Authors:** Shengjie Zhou, Yuxuan Qi, Ruifang Yang, Jing Yuan, Xian Cai, Wenxue Zhang, Yu Tian, Fei Ma

**Affiliations:** 1 Department of Epidemiology and Health Statistics, School of Public Health, Tianjin Medical University, Tianjin 300070, China; 2 Key Laboratory of Prevention and Control of Major Diseases in the Population, Ministry of Education, Tianjin Medical University, Tianjin 300070, China; 3 Department of Clinical Laboratory, Tianjin Medical University General Hospital, Tianjin 300052, China

**Keywords:** cMIND, cognitive decline trajectories, psychological resilience, CLHLS

## Abstract

Background: The Chinese version of the Mediterranean–DASH (Dietary Approaches to Stop Hypertension) Intervention for Neurodegenerative Delay (cMIND) diet is hypothesized to slow cognitive decline; nevertheless, most existing evidence is cross-sectional, precluding examination of the longitudinal association between the cMIND diet and heterogeneous cognitive decline trajectories. Moreover, it remains unclear whether psychological resilience mediates this relationship in older Chinese adults. Objectives: We assessed the longitudinal association between cMIND diet adherence and dynamic cognitive trajectories, and evaluated whether psychological resilience mediated the association between the cMIND diet and cognitive trajectories. Methods: Data came from the Chinese Longitudinal Healthy Longevity Survey (CLHLS), a national survey of older adults. cMIND diet scores were grouped into lower (0–4), middle (4.5–5.5), and higher (6–12). A group-based trajectory model (GBTM) was used to identify cognitive decline trajectories. A multinomial logistic regression model was applied to analyse the relationship between the cMIND diet pattern and cognitive decline trajectories in older adults. A mediation analysis was performed on the potential mediating effect of psychological resilience. Results: A total of 1755 participants were included. Participants were divided into three trajectories: the low-level rapid decline trajectory (*n* = 344, 19.6%), the high-level stable trajectory (*n* = 551, 31.4%), and the moderate-level slow decline trajectory (*n* = 860, 49.0%). Compared with the stable trajectory, lower diet scores were associated with the rapid decline cognitive trajectory (*OR*: 1.59; 95% *CI*: 1.03, 2.48) and the slow decline cognitive trajectory (*OR*: 1.61; 95% *CI*: 1.16, 2.23). The mediating effect of psychological resilience on the relationship between the cMIND diet pattern and cognitive trajectories was not statistically significant (*p* > 0.05). Conclusions: The cMIND diet pattern was significantly associated with cognitive trajectories in older Chinese adults, with higher adherence associated with stable trajectories and lower adherence associated with rapid and slow decline trajectories. However, psychological resilience did not significantly mediate the relationship between the cMIND diet pattern and cognitive trajectories.

## 1. Introduction

As the global population ages, cognitive decline and related neurodegenerative diseases have emerged as major public health challenges. An estimated 57.4 million people worldwide were living with dementia in 2019, and this number is projected to rise to 152.8 million by 2050 [1]. China, home to the world’s largest older population, bears a particularly heavy burden of cognitive impairment. Among Chinese persons 60 years and older, the prevalence rates of mild cognitive impairment (MCI) and dementia are 15.5% and 6.0%, respectively [2]. Cognitive decline is a dynamic process [3]. However, this progression is not uniform, and individuals exhibit substantial heterogeneity in their cognitive trajectories, ranging from long-term stability to slow or rapid decline [4]. Identifying modifiable risk factors to delay adverse cognitive trajectories is therefore crucial for promoting healthy aging.

Among the various modifiable factors, a healthy dietary pattern is considered a key strategy for slowing cognitive decline. Unlike single nutrients, overall dietary patterns better capture the synergistic effects among different foods. The Mediterranean-DASH (Dietary Approaches to Stop Hypertension) Intervention for Neurodegenerative Delay (MIND) diet has demonstrated significant neuroprotective effects in multiple Western studies. Greater adherence to the MIND diet has been associated with better cognitive function, slower cognitive decline, and a reduced risk of dementia [5,6]. However, the MIND diet is rooted in Western dietary habits—such as an emphasis on olive oil and wine—which differ considerably from traditional Chinese dietary patterns characterized by plant-based foods and whole-grain consumption. This cultural gap may limit its direct applicability to Chinese populations.

In response, researchers have developed a culturally adapted version of the MIND diet tailored to the Chinese population, termed the Chinese version of the Mediterranean–DASH (Dietary Approaches to Stop Hypertension) Intervention for Neurodegenerative Delay (cMIND) diet. This modified pattern retains core neuroprotective components (e.g., fruits, vegetables, and nuts) while incorporating adaptations based on Chinese dietary guidelines [7]. Preliminary cross-sectional studies have shown that higher cMIND diet scores are associated with a lower risk of cognitive impairment among older Chinese adults [7,8], providing initial evidence for its potential benefits. Nevertheless, cross-sectional designs cannot elucidate the longitudinal relationship between the cMIND diet and dynamic cognitive trajectories, nor can they adequately reveal the underlying mechanisms.

In recent years, psychological resilience—defined as an individual’s ability to adapt positively and maintain sound psychological functioning in the face of stress, adversity, or trauma—has gained increasing attention as a vital psychosocial resource in the field of cognitive aging [9]. Converging theoretical and empirical evidence suggests that higher levels of psychological resilience are associated with better cognitive function and slower cognitive decline [10,11]. Psychological resilience may promote cognitive health through multiple pathways, including buffering the detrimental effects of chronic stress on the brain and encouraging health-promoting behaviors (e.g., maintaining a healthy diet and engaging in cognitively stimulating activities) [12,13]. It may also influence cognition by fostering positive emotion regulation and adaptive coping strategies [14].

Notably, emerging evidence indicates an association between dietary patterns and psychological resilience. For example, lower dietary diversity has been linked to reduced psychological resilience in older adults [15]. Conversely, healthy dietary patterns rich in antioxidants and anti-inflammatory compounds, such as the Mediterranean diet, may enhance psychological resilience by mitigating oxidative stress and neuroinflammation [16,17]. These findings provide theoretical support for a potential mediating pathway linking diet, psychological resilience, and cognitive health.

To date, however, few studies have examined the potential mediating role of psychological resilience in the relationship between the cMIND diet and cognitive decline trajectories among older Chinese adults. Specifically, it remains unknown whether the cMIND diet influences cognitive trajectories by affecting individuals’ levels of psychological resilience. Elucidating this mediating mechanism carries substantial theoretical and practical importance: it would not only deepen our understanding of how the cMIND diet exerts its cognitive-protective effects, but also identify dietary patterns and psychological resilience as potential targets for future cognitive health research.

## 2. Materials and Methods

### 2.1. Study Population

This study used data from the Chinese Longitudinal Healthy Longevity Survey (CLHLS), a nationwide prospective cohort study of Chinese persons 65 years and older. Following the baseline survey in 1998, eight follow-up surveys were conducted in 2000, 2002, 2005, 2008–2009, 2011–2012, 2014, and 2017–2018. The present study drew on data from the 2008 wave (baseline) and subsequent follow-ups in 2011, 2014, and 2018, initially including 16,954 older adults. All participants provided written informed consent before enrollment, and the study was approved by the Biomedical Ethics Committee of Peking University (IRB00001052-13074).

**Figure 1:**
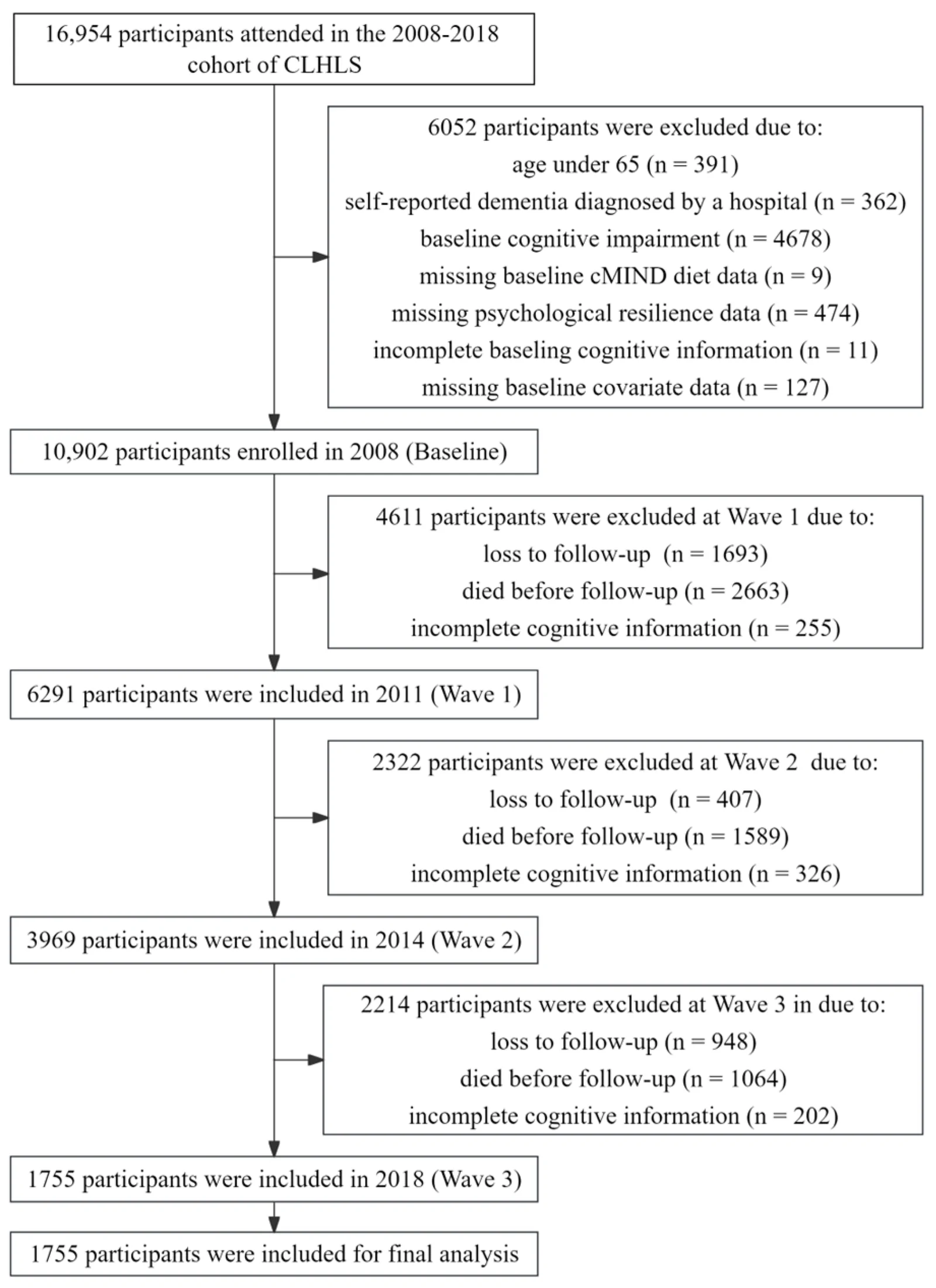
Flowchart demonstrating the participant selection process. cMIND, the Chinese version of the Mediterranean–DASH (Dietary Approaches to Stop Hypertension) Intervention for Neurodegenerative Delay.

Participants were excluded according to the following criteria: age under 65 years; presence of cognitive impairment at baseline [18]; self-reported dementia diagnosed by a hospital at baseline; incomplete data on cognitive function, cMIND diet, or psychological resilience at baseline; and failure to complete the Mini-Mental State Examination (MMSE) assessment at any of the four longitudinal follow-ups. After applying these exclusion criteria, a total of 1755 participants were included in the final analysis. Comparison of baseline characteristics between included (*n* = 1755) and excluded (*n* = 15,199) participants (Supplementary Table S1) revealed statistically significant differences for all variables except the presence of chronic diseases. The study flow chart is presented in Figure 1.

### 2.2. cMIND Diet Pattern Assessment

The cMIND diet scale was developed for the Chinese population based on the original MIND diet scale and the CLHLS food frequency questionnaire, and its reliability has been evaluated in this population [7]. The cMIND diet comprises 12 food components: staple food composition, staple food quantity, fresh fruits, fresh vegetables, cooking oil, fish, bean-based foods, sugar and tea, garlic, nuts, and fungi/algae. The total cMIND score ranges from 0 to 12 points. A score of 1 was assigned if whole grains constituted the staple food, if daily staple food intake was within 250–400 g, or if vegetable oil was used (tropical oils were not distinguished from other vegetable oils, as such categorization was not available in the CLHLS questionnaire); otherwise, a score of 0 was given. The remaining food items were scored based on consumption frequency, categorized as “almost every day,” “≥once per week,” “≥once per month,” “occasionally,” or “rarely or never,” with corresponding scores of 0, 0.5, or 1. Specifically, higher consumption frequencies of fresh fruits, fresh vegetables, fish, bean-based foods, tea, garlic, nuts, and fungi/algae were associated with higher scores, whereas a higher consumption frequency of sugar was associated with a lower score. cMIND diet scores were grouped into lower (0–4), middle (4.5–5.5), and higher (6–12) according to the existing cMIND study [19,20]. Detailed scoring criteria are provided in Table S2.

### 2.3. Cognitive Function Assessment

Cognitive function was assessed using the Chinese Mini-Mental State Examination (cMMSE) with a total possible score of 30, comprising 15 items across orientation, registration, attention and calculation, recall, and language domains [21]. Each correctly answered item receives 1 point; incorrect or missing responses receive 0 points, with higher scores indicating better cognitive function. Given the potential influence of education on cMMSE performance, cognitive impairment was defined using education-adjusted cut-offs: ≤18 for no formal education, ≤21 for 1–6 years, and ≤25 for more than 6 years [18]. The validity and reliability of the cMMSE have been well established in previous studies (Table S3) [22,23].

Trajectory models were estimated for one to five groups, testing polynomial orders up to cubic (highest order = 3) to capture potential nonlinear patterns. The optimal model was selected based on the following criteria: minimization of information criteria (AIC, BIC, CAIC, and SABIC), average posterior probability (AvePP > 0.7), odds of correct classification (OCCs > 5), class proportion (prior > 15%), and distinctiveness of trajectories.

### 2.4. Psychological Resilience Assessment

Self-reported psychological resilience was assessed using the Simplified Resilience Scale (SRS), which is based on seven resilience-related questions from the CLHLS: “Can you manage to keep a positive outlook no matter what happens?” “Do you like to keep things clean and tidy?” “Can you make your own decisions about your affairs?” “Do you feel as cheerful as you did when you were younger?” “Do you often feel anxious or afraid?” “Do you often feel lonely?” and “Do you feel that you are becoming less capable as you get older?” These seven items reflect perseverance, optimism, the ability to cope with difficulties, stable interpersonal relationships, and self-control. Each question offers six response options: always, often, sometimes, seldom, never, and unable to answer. Items 1, 2, 5, and 7 were reverse-scored. For these items, response options 1 through 5 were assigned scores of 5, 4, 3, 2, and 1, respectively. Items 3, 4, and 6 were scored directly, with responses 1 to 5 corresponding to 1 to 5 points. Responses marked as “unable to answer” were treated as missing data. The total SRS score, ranging from 5 to 35, was calculated by summing the scores of all seven items, with higher scores indicating greater resilience. Several previous studies have used all or some of these seven items to measure resilience [24,25,26], indicating that the SRS based on CLHLS data is reasonably acceptable (Table S4).

### 2.5. Covariate Assessment

Based on the existing literature [27], models were adjusted for demographic covariates including age, sex, years of schooling (0/1–6/>6), living arrangement (living with family/alone/nursing home), residence (urban/rural), marital status (married/divorced/widowed/never married), and economic status (poor/general/rich), health-related covariates including body mass index (BMI: underweight (<18.5)/normal (18.5–23.9)/overweight (24.0–27.9)/obese (≥28.0) kg/m^2^) [28], vitamin A, C, and E supplementation (yes if taken at least once per month, otherwise no; vitamin supplementation was limited to vitamins A, C, and E, as the CLHLS questionnaire assessed these three vitamins as a single grouped item and lacked data on other vitamins), exercise (yes/no), drinking (yes/no), smoking (yes/no), and chronic diseases (hypertension, heart disease, cerebrovascular disease, cataract, lung disease, cancer, Parkinson’s disease, arthritis, blood disease, and diabetes), classified as present or absent.

### 2.6. Statistical Analysis

Group-based trajectory modeling (GBTM) was used to identify distinct cognitive decline trajectory groups. Descriptive statistics were calculated according to the trajectory groups to compare baseline characteristics. Continuous psychological resilience scores were used in the mediation analysis to enhance statistical power. Continuous variables were presented as mean ± standard deviation ($\bar{x}$ ± SD), while non-normally distributed continuous variables were presented as medians with interquartile ranges (median [IQR]). Categorical variables were expressed as frequencies and percentages (n [%]). The Shapiro–Wilk test was used to assess normality. ANOVA, the Kruskal–Wallis H test, and R × C chi-square tests were used to compare baseline characteristics across trajectory groups.

To examine the association between the cMIND diet pattern and cognitive decline trajectories, multinomial logistic regression models were applied. The cognitive trajectory categories served as the dependent variable (with the stable trajectory as the reference), and the cMIND diet score served as the independent variable (with the high diet score group as the reference). Three models were constructed: a crude model without covariates, a model adjusted for demographic covariates, and a fully adjusted model including all covariates. Statistical significance was set at *p* < 0.05. Data cleaning was performed using Stata version 18.0 (StataCorp, College Station, TX, USA), statistical analyses were conducted using IBM SPSS Statistics version 27.0 (IBM Corp., Armonk, NY, USA), trajectory modeling was performed using the gbmt package in R 4.5.1, and mediation analysis using the bootstrap approach was conducted with the lavaan package in R.

Sensitivity analyses were conducted to assess the robustness of the associations among the cMIND diet pattern, psychological resilience, and cognitive decline trajectories. First, the sample was restricted to participants with MMSE data at a minimum of three time points: specifically, at baseline (2008) and first follow-up (2011), as well as at either the 2014 or 2018 wave. Second, to examine whether the classification of chronic conditions influenced the observed associations, the single chronic disease variable was replaced with two separate variables, each classified as present or absent: (1) cardiometabolic and neurodegenerative diseases (hypertension, diabetes, heart disease, cerebrovascular disease, and Parkinson’s disease), and (2) other chronic diseases (cataract, lung disease, cancer, arthritis, and blood disease), both of which were simultaneously entered into the model as covariates.

## 3. Results

### 3.1. Baseline Characteristics

A total of 1755 older adults were included in this study. The median age was 73.00 years, 52.8% of participants were female, and 48.1% were illiterate. The majority of participants were married (62.4%), lived with family members (84.1%), resided in rural areas (86.9%), and reported general economic status (72.3%). In terms of health behaviors, 77.3% were non-smokers, 77.7% were non-drinkers, 64.6% did not engage in regular exercise, 65.0% had a normal BMI, 86.3% reported not taking vitamin supplements, and 52.6% had chronic diseases. For the cMIND diet score, 27.7% of participants had a lower score, 39.4% had a moderate score, and 32.9% had a higher score.

**Figure 2:**
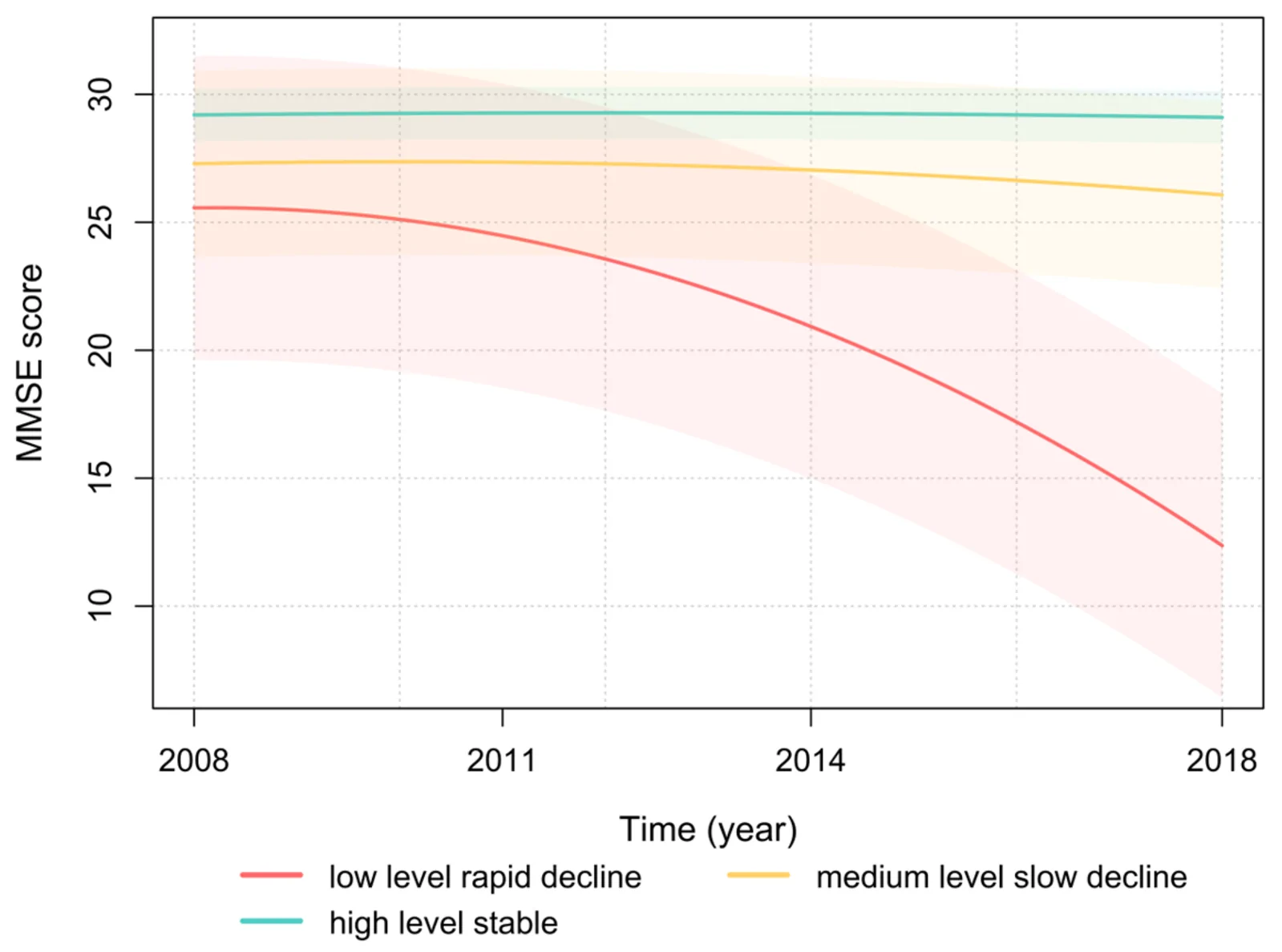
Three cognitive trajectories. The figure shows the decline in cMMSE score between 2008 and 2018. Three distinct cognitive decline trajectories were defined as the low-level rapid decline group, the moderate-level slow decline group, and the high-level stable group. The study used the high-level stable group (green) as a reference. The cMMSE score ranges from 0 to 30 points. cMMSE, Chinese Mini-Mental State Examination.

Based on a comprehensive assessment of statistical indicators (AvePP: 0.96/0.93/0.94, OCCs: 89.26/31.50/17.68), model interpretability, group reliability, and parsimony, a three-class model was selected as the optimal representation of cognitive trajectories, which were defined as the low-level rapid decline group (*n* = 344, 19.6%), the moderate-level slow decline group (*n* = 860, 49.0%), and the high-level stable group (*n* = 551, 31.4%). The developmental trends of each trajectory are shown in Figure 2 (detailed scoring criteria are presented in Table S5; one-, two-, four-, and five-group trajectory model plots are shown in Supplementary Figures S1–S4).

Comparison of baseline characteristics across the three trajectory groups revealed statistical differences for all variables except vitamin supplementation and presence of chronic diseases (*p* < 0.05). Baseline characteristics results are presented in Table 1.

### 3.2. Association Between the cMIND Diet Pattern and Cognitive Trajectories

Logistic regression analyses examining the association between the cMIND diet and cognitive decline trajectories showed that, in the unadjusted model (Model 1), compared with the high-level stable trajectory group, lower and middle cMIND diet scores were associated with the rapid decline of cognitive trajectories and the slow decline of cognitive trajectories. After adjusting for sociodemographic covariates (Model 2), the associations of the lower diet score with the rapid decline (*OR*: 1.64; 95% *CI*: 1.06, 2.53) and slow decline (*OR*: 1.64; 95% *CI*: 1.19, 2.26) trajectories remained significant, although the effect sizes were attenuated.

Further adjustment for all covariates (Model 3) showed that the lower diet score continued to be associated with both the rapid decline trajectory (*OR*: 1.59; 95% *CI*: 1.03, 2.48) and the slow decline (*OR*: 1.61; 95% *CI*: 1.16, 2.23) trajectory, with further reduction in the magnitude of associations. In contrast, although a middle diet score was associated with both cognitive decline trajectories in Model 1, these associations became non-significant in Models 2 and 3 (Table 2). Further analysis revealed that, after adjusting for all covariates, consumption of fresh fruits was associated with lower odds of the rapid decline trajectory compared to the stable trajectory (*OR*: 0.64; 95% *CI*: 0.42, 0.97). However, no statistically significant associations were observed for the remaining 11 cMIND components (whole grains, staple quantity, vegetables, plant oil, fish, beans, sugar, tea, garlic, nuts, and algae/mushrooms) with either the rapid or slow decline trajectory (all *p* > 0.05; refer to Table S6).

### 3.3. Association Between Covariates and Cognitive Trajectories

Logistic regression analyses examining covariates and cognitive trajectories revealed that older age, female sex, and no formal schooling were associated with both the rapid and the slow cognitive decline trajectories compared with the stable trajectory. Moreover, participants with normal weight had lower odds of being in the rapid decline trajectory than those with obesity. Complete results are presented in Table S7.

### 3.4. Association Between the cMIND Diet and Psychological Resilience

Multivariate linear regression analysis demonstrated that the standardized *β* coefficients for cMIND diet scores were positive across all three models (0.232, 0.143, and 0.125; all *p* < 0.001). This indicates that, after adjusting for other factors, each one-point increase in the cMIND diet score was associated with an expected increase of approximately 0.125 points in the psychological resilience score, suggesting a positive association between cMIND diet and psychological resilience (Table 3).

### 3.5. Association Between Psychological Resilience and Cognitive Trajectories

Logistic regression analyses of the relationship between psychological resilience and cognitive trajectories revealed that, compared with the high-level stable group, higher psychological resilience was negatively associated with the probability of belonging to both the rapid and the slow decline trajectories. After adjusting for all covariates, each one-point increase in the resilience score was associated with approximately 9.0% lower odds of being classified into the rapid decline trajectory (*OR*: 0.91; 95% *CI*: 0.87, 0.95) and approximately 4.1% lower odds of belonging to the slow decline trajectory (*OR*: 0.96; 95% *CI*: 0.93, 0.94). These significant protective associations remained consistent across the unadjusted, partially adjusted, and fully adjusted models (Table 4).

### 3.6. Mediation Analysis of Psychological Resilience on the cMIND Diet and Cognitive Trajectories

**Figure 3:**
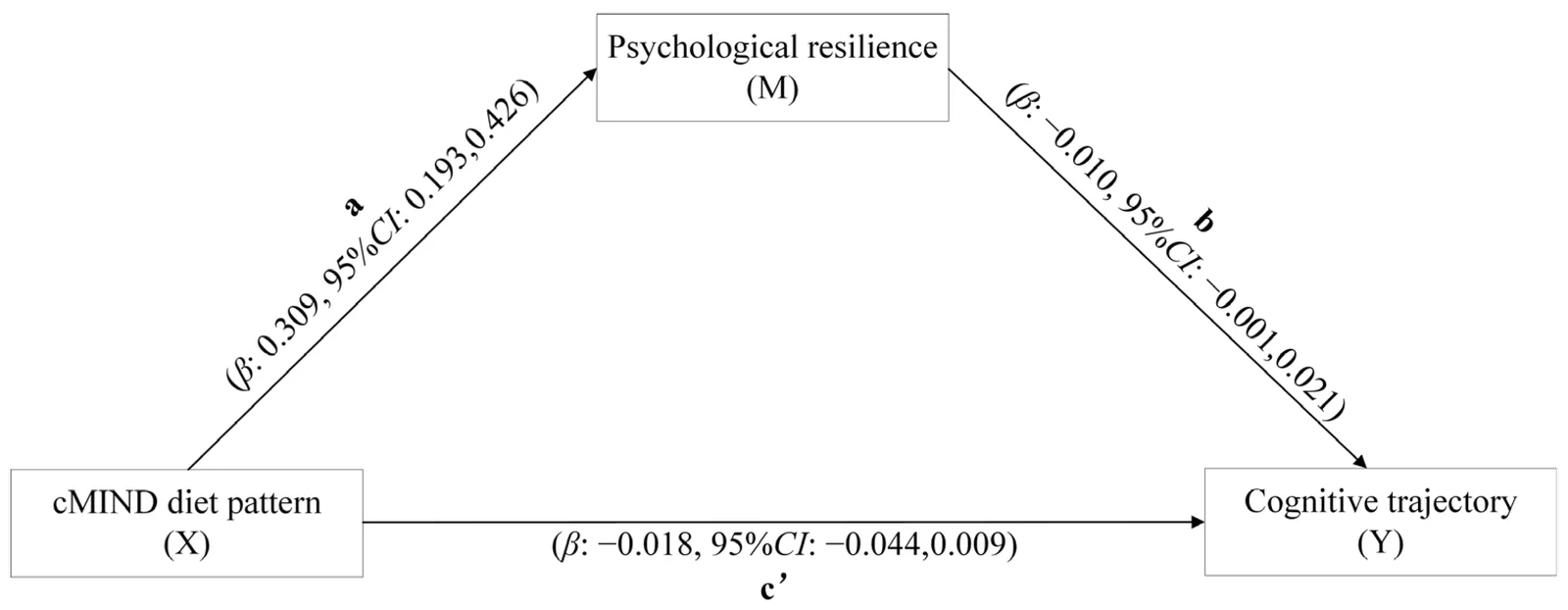
Mediation analysis diagram. **a** represents the effect of the independent cMIND diet on psychological resilience; **b** represents the effect of the independent psychological resilience on the cognitive trajectory; **c’** represents the direct effect of the cMIND diet on cognitive trajectories.

The mediation analysis indicated that the mediating effect of psychological resilience on the relationship between the cMIND diet and cognitive trajectories was not statistically significant (Figure 3). Specifically, the cMIND diet pattern showed a significant positive predictive effect on psychological resilience (*β*: 0.31; 95% *CI*: 0.19, 0.43). However, the predictive effect of psychological resilience on cognitive trajectories was not significant (*β*: 0.01; 95% *CI*: 0.00, 0.02). The estimated coefficient of the indirect effect was 0.00, with a bootstrap 95% confidence interval of −0.01 to 0.00 (Table 5).

### 3.7. Sensitivity Analysis

The results of the sensitivity analysis were consistent with the main analysis. The first sensitivity analysis included a total of 4184 participants (Table S8). The association between the lower cMIND diet score and trajectories of rapid (*OR*: 1.53; 95% *CI*: 1.16, 2.02) and slow cognitive decline (*OR*: 1.44; 95% *CI*: 1.15, 1.79) remained significant after adjusting for all covariates (Table S9). Logistic regression analyses examining covariates and cognitive trajectory revealed that, in addition to age, sex, and years of schooling, poor economic status was also associated with cognitive decline trajectories (Table S10). Mediation analysis (Table S11) further supported that psychological resilience did not mediate the relationship between the cMIND diet and cognitive trajectories (*β*: 0.00; 95% *CI*: 0.00, 0.00) [29].

In the second sensitivity analysis, where chronic conditions were reclassified into cardiometabolic and neurodegenerative diseases versus other chronic diseases (Table S12), the results remained robust and consistent with the main analysis. The association between the lower cMIND diet score and trajectories of rapid (*OR*: 1.60; 95% *CI*: 1.03, 2.49) and slow cognitive decline (*OR*: 1.63; 95% *CI*: 1.17, 2.25) remained significant after full adjustment (Table S13). Logistic regression analyses examining covariates and cognitive trajectory revealed that age, sex, and years of schooling were associated with cognitive decline trajectories (Table S14). Multivariate linear regression analysis demonstrated a positive association between the cMIND diet and psychological resilience (standardized *β*: 0.232, 0.143, and 0.126; all *p* < 0.001), consistent with the main analysis (Table S15). Logistic regression analyses of the relationship between psychological resilience and cognitive trajectories revealed that, compared with the high-level stable group, higher psychological resilience was negatively associated with the probability of belonging to both the rapid (*OR*: 0.91; 95% *CI*: 0.87, 0.96) and slow decline trajectories (*OR*: 0.96; 95% *CI*: 0.93, 0.99), consistent with the main analysis (Table S16). Consistently, mediation analysis (Table S17) further confirmed that psychological resilience did not mediate the relationship between the cMIND diet and cognitive trajectories (*β*: 0.00; 95% *CI*: 0.00, 0.01).

## 4. Discussion

This study used longitudinal data from the CLHLS to investigate the associations among the cMIND diet pattern, psychological resilience, and heterogeneous cognitive decline trajectories in older Chinese adults. The key findings are fourfold. First, using GBTM analysis, three distinct cognitive trajectories were identified over time: high-level stable, moderate-level slow decline, and low-level rapid decline. Second, a lower cMIND diet score was associated with both the rapid decline and slow decline trajectories compared with the stable trajectory, after covariate adjustment. Third, a positive cross-sectional association was observed between the cMIND diet and psychological resilience. Fourth, and notably, the mediation analysis did not support a significant indirect effect of psychological resilience on the association between the cMIND diet and cognitive trajectories.

The three distinct cognitive trajectories identified in this study align with findings from other cohort studies in China and internationally [4,30]. This confirms that older adults are not homogeneous in their cognitive aging process, and tools such as trajectory modeling are crucial for identifying high-risk subgroups—such as those in the low-level rapid decline trajectory group—who represent the subgroups most in need of attention. Furthermore, this study provides longitudinal evidence supporting the cognitive health benefits of the cMIND diet for older adults in China. The findings are consistent with the MIND diet research by Morris et al. [5] and the conclusions of the review by van den Brink [6], indicating that the MIND diet effectively slows cognitive decline [31]. Specifically, this study’s results not only corroborate the cross-sectional findings of Huang et al. [7] but also add a dynamic dimension to them. As older adults without cognitive impairment at baseline were followed over time, the possibility of reverse causality is reduced [6], and selection bias is mitigated [5]. Moreover, this study extends the longitudinal analysis of Lin et al. [8]. While Lin et al. demonstrated an association between the cMIND diet and better cognitive function at a single time point, the present study, by applying trajectory modeling [32] to multiple follow-up waves of cognitive assessments, further shows that higher cMIND adherence is associated with a stable cognitive trajectory over time.

Several components of the cMIND diet represent culturally tailored modifications to the original MIND diet, which merit further discussion. First, regarding staple foods, the cMIND diet evaluates both the type and the total amount of staple food consumed. Given that both excessive and insufficient carbohydrate intake have been shown to impair cognitive function, moderate consumption of staple foods may be more appropriate for older adults [33]. Second, in terms of cooking oil, the original MIND diet specifies olive oil. However, olive oil is rarely used in Chinese households; therefore, the cMIND diet scores vegetable oil versus animal oil. Vegetable oil is characterized by higher levels of monounsaturated and polyunsaturated fatty acids, a fatty acid profile that is more favorable for maintaining cognitive function [33]. Third, consumption of mushrooms and algae is included as an independent component in the cMIND diet. Observational evidence has linked higher mushroom intake to better cognitive performance and a lower risk of mild cognitive impairment and dementia [34,35], while seaweed-derived compounds have been investigated for their acute postprandial cognitive effects [36]. The potential neuroprotective properties of mushrooms and algae are attributed to their bioactive polysaccharides, trace elements, and anti-inflammatory phytochemicals rather than to conventional nutrient composition alone [37,38]. Fourth, garlic is included in the cMIND diet but not in the original MIND diet, given its frequent use in Chinese cooking. Evidence from animal models indicates that garlic extract mitigates Alzheimer’s disease pathology by decreasing amyloid-*β* accumulation and alleviating oxidative injury and neuroinflammation [39,40]. Fifth, given the distinct alcohol consumption patterns among older Chinese adults and their prevalent tea-drinking habit, the original MIND diet’s daily wine recommendation was replaced with tea in the cMIND diet. Prospective studies have shown that higher tea consumption is associated with a reduced risk of cognitive decline [41], an effect primarily attributed to catechins, which exhibit antioxidant and anti-neuroinflammatory properties [42].

Beyond individual food items, the fatty acid composition embedded in the cMIND diet warrants further discussion. Monounsaturated fatty acids (MUFAs), abundant in vegetable oil and nuts, improve vascular function and maintain neuronal membrane fluidity [43,44]. Polyunsaturated fatty acids (PUFAs), particularly *n*-3 PUFAs such as eicosapentaenoic acid (EPA) and docosahexaenoic acid (DHA) from fish, play critical roles in anti-inflammatory signaling, myelination, and synaptic transmission [45,46,47]. Plant-derived *n*-6 PUFAs, when consumed in moderation, contribute to cognitive maintenance, although an imbalanced *n*-6: *n*-3 ratio may promote pro-inflammatory effects [48,49].

The combined effects of these fatty acids, together with other bioactive components from vegetables, fruits, mushrooms, garlic, and tea, may provide biological plausibility for the association between the cMIND diet and cognitive trajectories [50]. The plausibility of this association is supported by mechanisms including reduced oxidative stress and inflammation, as well as modulation of the gut–brain axis. Specifically, based on the current literature, the cMIND diet provides antioxidants and polyphenols that mitigate oxidative stress and inhibit pro-inflammatory pathways, thereby reducing neuroinflammation [1,5,51]. Concurrently, its high fiber content acts as a prebiotic, fostering a beneficial gut microbiota that produces anti-inflammatory short-chain fatty acids (SCFAs) [6,7]. SCFAs help maintain gut barrier integrity, prevent endotoxin translocation, and have been shown to modulate neuroinflammation and neuroplasticity via systemic circulation and vagal pathways, thereby influencing the gut–brain axis [6,7,8,9,51,52,53]. Thus, the effect of the cMIND diet on cognitive trajectories likely arises from an integrated model, consistent with the mechanisms proposed for the original MIND diet [6,16].

Component-level analysis revealed that, among the 12 cMIND components, only the consumption of fresh fruits was independently associated with lower odds of the rapid decline trajectory after full adjustment. This result corroborates prior CLHLS-based evidence [8]. The fact that several components were non-significant in isolation does not contradict the association observed for the cMIND dietary pattern. Rather than reflecting the effect of a single food, the observed cognitive benefits appear to emerge from the synergistic interactions of multiple cMIND components. These results therefore argue for prioritizing whole-diet approaches over isolated nutrients when mitigating cognitive decline in older adults [50].

The observed positive association between cMIND diet adherence and psychological resilience is a novel and important finding. It aligns with and expands upon prior studies linking dietary diversity [15] and Mediterranean-style dietary patterns [17] to better psychological resilience. This suggests that the cMIND diet, rich in neuroprotective components, may be positively associated not only with cognitive function but also with greater psychological resilience in older adults.

Two distinct, non-mutually exclusive interpretations for the non-significant indirect pathway in the mediation analysis warrant consideration. First, from a methodological perspective, the lack of a significant mediation effect may relate to statistical power. Although psychological resilience may indeed play a mediating role, given the modest a-path standardized *β* (*β*: 0.125) and b-path *OR* (*OR*: 0.91, log *OR*: −0.094), the indirect effect (a × b: −0.003) was indeed small. According to Fritz and MacKinnon, detecting such an effect with 0.80 power requires substantially larger sample sizes [54]. Therefore, both our primary and sensitivity samples may be underpowered. Second, the cMIND diet and psychological resilience may confer cognitive benefits through largely independent mechanisms: the former potentially via direct biological pathways (e.g., nutrient supply, anti-inflammatory actions) [5,6], and the latter via distinct psychosocial mechanisms (e.g., stress buffering, maintenance of social engagement) [9,13,51,55]. Consequently, while resilience may not serve as a mediator, future research could explore its potential as a moderator by examining whether the strength of the association between the cMIND diet and cognitive outcomes varies across levels of psychological resilience.

The findings underscore the value of the cMIND dietary pattern for cognitive health [7]. For high-risk populations—such as older adults, females, and individuals with no formal schooling, who are more likely to follow adverse “rapid decline” or “slow decline” cognitive trajectories—greater attention to dietary quality may be particularly important. More importantly, our results suggest that the relevance of dietary advice may vary by adherence level. For individuals with a lower cMIND diet score, improving overall dietary quality may offer the greatest potential benefit. However, for those with a middle diet score, whose cognitive risk appears to be primarily influenced by broader confounding factors, a broader range of factors needs to be considered to delay cognitive decline.

This study has several notable strengths. First, it utilizes a large, nationally representative longitudinal cohort of older Chinese persons ≥ 65 years, ensuring generalizability. Second, the application of GBTM went beyond traditional static analyses to identify distinct, heterogeneous trajectories of cognitive decline.

However, several limitations should be acknowledged. First, although a comprehensive set of baseline covariates was adjusted for, residual confounding from unmeasured factors cannot be entirely ruled out. Second, this study explored the association between participants’ baseline cMIND diet pattern and cognitive decline trajectories. However, assessments of psychological resilience and dietary status may have changed over time, suggesting that their effects on cognition are likely dynamic. Therefore, further research is needed to investigate the impact of time-varying cMIND diet patterns on cognitive function. Third, methodological limitations associated with applying structural equation modeling (via the lavaan package) to a three-category outcome should be acknowledged. While lavaan provides a flexible framework for testing mediation models, its standard estimation is primarily designed for continuous outcomes. When applied to a categorical dependent variable, the model estimates and significance tests for indirect effects may not be fully appropriate [56]. Fourth, selection bias due to loss to follow-up and death should be considered. Comparison of baseline characteristics between included and excluded participants revealed statistical differences for all variables except the presence of chronic diseases. This systematic attrition may introduce bias into the estimated association between cMIND and cognition. Although sensitivity analyses using multiple imputation yielded consistent results, residual selection bias cannot be fully excluded. Fifth, baseline dementia status was based on self-reported dementia diagnosed by a hospital rather than independent verification via medical records, which may introduce bias. Sixth, the current cMIND diet does not adequately differentiate among various types of vegetable oils in terms of their potential effects on cognitive health. Tropical oils and other vegetable oils differ substantially in their fatty acid profiles, which may lead to divergent impacts on cognitive function [57,58]. Consequently, broadly attributing health benefits to vegetable oils as a single category remains a notable limitation. Future studies should adopt a more refined classification of vegetable oils within the cMIND framework to determine whether the cognitive benefits associated with this dietary pattern vary depending on the specific types of vegetable oils consumed.

## 5. Conclusions

The cMIND diet pattern was significantly associated with cognitive trajectories in older Chinese adults, with higher adherence associated with stable trajectories and lower adherence associated with rapid and slow decline trajectories. However, psychological resilience did not significantly mediate this association. These findings may offer novel insights into the potential benefits of the cMIND diet for cognitive function, particularly among older adults, females, and individuals with no formal schooling.

## Figures and Tables

**Table 1:** Baseline characteristics of the overall sample and different trajectory groups.

Variable	Category	Total Sample (*n* = 1755)	Trajectory Groups			*H*/*X*^2^	*p*
			Rapid-Decline (*n* = 344)	Stable (*n* = 551)	Slow-Decline (*n* = 860)		
Age		73.0 (69.0, 80.0)	80.5 ± 7.8	70.0 (67.0, 74.0)	73.0 (69.0, 79.0)	328.22	<0.001
Gender	male	828 (47.2)	105 (30.5)	345 (62.6)	378 (44.0)	94.56	<0.01
	female	927 (52.8)	239 (69.5)	206 (37.4)	482 (56.0)		
Educational attainment	low	844 (48.1)	253 (73.5)	127 (23.0)	464 (54.0)	282.03	<0.01
	medium	674 (38.4)	83 (24.1)	274 (49.7)	317 (36.9)		
	high	237 (13.5)	8 (2.3)	150 (27.2)	79 (9.2)		
Marital status	married	1095 (62.4)	147 (42.7)	419 (76.0)	529 (61.5)		<0.01
	divorced	3 (0.2)	0 (0.0)	3 (0.5)	0 (0.0)		
	widowed	642 (36.6)	195 (56.7)	125 (22.7)	322 (37.4)		
	single	15 (0.9)	2 (0.6)	4 (0.7)	9 (1.0)		
Living situation	with family	1476 (84.1)	267 (77.6)	496 (90.0)	713 (82.9)		<0.01
	living alone	269 (15.3)	75 (21.8)	51 (9.3)	143 (16.6)		
	nursing home	10 (0.6)	2 (0.6)	4 (0.7)	4 (0.5)		
Residence	rural	1525 (86.9)	311 (90.4)	454 (82.4)	760 (88.4)	15.17	<0.01
	urban	230 (13.1)	33 (9.6)	97 (17.6)	100 (11.6)		
Economic status	good	220 (12.5)	38 (11.0)	94 (17.1)	88 (10.2)	24.29	<0.01
	normal	1269 (72.3)	241 (70.1)	397 (72.1)	631 (73.4)		
	poor	266 (15.2)	65 (18.9)	60 (10.9)	141 (16.4)		
Smoking	yes	398 (22.7)	52 (15.1)	163 (29.6)	183 (21.3)	27.16	<0.01
	no	1357 (77.3)	292 (84.9)	388 (70.4)	677 (78.7)		
Drinking	yes	391 (22.3)	58 (16.9)	152 (27.6)	181 (21.0)	15.55	<0.01
	no	1364 (77.7)	286 (83.1)	399 (72.4)	679 (79.0)		
Exercise	yes	622 (35.4)	97 (28.2)	220 (39.9)	305 (35.5)	12.74	0.002
	no	1133 (64.6)	247 (71.8)	331 (60.1)	555 (64.5)		
BMI (kg/m^2^)	underweight	366 (20.9)	102 (29.7)	75 (13.6)	189 (22.0)	45.46	<0.001
	normal	1140 (65.0)	197 (57.3)	375 (68.1)	568 (66.0)		
	overweight	213 (12.1)	34 (9.9)	89 (16.2)	90 (10.5)		
	Obesity	36 (2.1)	11 (3.2)	12 (2.2)	13 (1.5)		
Vitamin A, C, and E supplementation	yes	191 (10.9)	35 (10.2)	71 (12.9)	85 (9.9)	3.34	0.188
	no	1564 (89.1)	309 (89.8)	480 (87.1)	775 (90.1)		
Chronic disease	yes	924 (52.6)	170 (49.4)	286 (51.9)	468 (54.4)	2.64	0.267
	no	831 (47.4)	174 (50.6)	265 (48.1)	392 (45.6)		
cMIND diet scorea		5.0 (4.0, 6.0)	5.0 (4.0, 6.0)	5.5 (4.5, 6.5)	5.0 (4.0, 6.0)	49.28	<0.001
cMIND diet group	lower	486 (27.7)	116 (33.7)	110 (20.0)	260 (30.2)	42.38	<0.01
	medium	692 (39.4)	138 (40.1)	207 (37.6)	347 (40.3)		
	higher	577 (32.9)	90 (26.2)	234 (42.5)	253 (29.4)		
SRS scoreb		26.0 (24.0, 29.0)	25.0 (23.0, 28.0)	28.0 (25.0, 30.0)	26.0 (24.0, 29.0)	87.63	<0.001

Continuous variables were presented as $\bar{x}$ ± SD or median (IQR), categorical variables as *n* (%). Group differences for continuous variables (Age, cMIND diet score, and SRS score) were assessed using the Kruskal–Wallis H test; group differences for the remaining categorical variables were assessed using R × C chi-square tests. Abbreviation: BMI, body mass index; cMIND, the Chinese version of the Mediterranean–DASH (Dietary Approaches to Stop Hypertension) Intervention for Neurodegenerative Delay; SRS, the Simplified Resilience Scale. The Chinese version of the Mediterranean–DASH (Dietary Approaches to Stop Hypertension) Intervention for Neurodegenerative Delay (cMIND) diet score ranges from 0 to 12. The Simplified Resilience Scale (SRS) score ranges from 5 to 35.

**Table 2:** Association between the cMIND diet pattern and cognitive trajectories.

Model	Diet Group	Rapid Decline vs. Stable			Slow Decline vs. Stable		
		*OR*	95% *CI*	*p*	*OR*	95% *CI*	*p*
Model 1	lower	2.74	1.92, 3.92	<0.001	2.19	1.64, 2.91	<0.001
	middle	1.73	1.25, 2.40	0.001	1.55	1.21, 1.99	0.001
	higher	1.00 (Ref)			1.00 (Ref)		
Model 2	lower	1.64	1.06, 2.53	0.025	1.64	1.19, 2.26	0.003
	middle	1.18	0.80, 1.75	0.402	1.24	0.94, 1.63	0.133
	higher	1.00 (Ref)			1.00 (Ref)		
Model 3	lower	1.59	1.03, 2.48	0.038	1.61	1.16, 2.23	0.004
	middle	1.15	0.77, 1.71	0.491	1.24	0.93, 1.64	0.140
	higher	1.00 (Ref)			1.00 (Ref)		

Model 1 did not include any covariate adjustments; Model 2 adjusted for Age, Gender, Years of schooling, Marital status, Living situation, Residence, and Economic status; Model 3 further adjusted for Smoking, Drinking, Exercise, BMI (kg/m^2^), Vitamin A, C, and E supplementation, and Chronic disease. Abbreviation: cMIND, the Chinese version of the Mediterranean–DASH (Dietary Approaches to Stop Hypertension) Intervention for Neurodegenerative Delay; *OR*, odds ratio; *CI*, confidence interval; Ref, reference category.

**Table 3:** Association between cMIND diet and psychological resilience.

Model	Unstandardised Coefficient *β*	Standardisation Coefficient *β*	t	95% *CI*	*p*	Statistics on Collinearity	
						Tolerance	VIF
Model 1	0.58	0.23	10.01	0.46, 0.69	<0.001	1.00	1.00
Model 2	0.35	0.14	6.03	0.24, 0.47	<0.001	0.86	1.16
Model 3	0.31	0.13	5.21	0.19, 0.43	<0.001	0.83	1.21

Model 1 did not include any covariate adjustments; Model 2 adjusted for Age, Gender, Educational attainment, Marital status, Living situation, Residence, and Economic status; Model 3 further adjusted for Smoking, Drinking, Exercise, BMI (kg/m^2^), Vitamin A, C, and E supplementation, and Chronic disease. Abbreviation: cMIND, the Chinese version of the Mediterranean–DASH (Dietary Approaches to Stop Hypertension) Intervention for Neurodegenerative Delay; *CI*, confidence interval; VIF, Variance Inflation Factor.

**Table 4:** Association between psychological resilience and cognitive trajectories.

Model	Rapid Decline vs. Stable			Slow Decline vs. Stable		
	*OR*	95% *CI*	*p*	*OR*	95% *CI*	*p*
Model 1	0.84	0.80, 0.87	<0.001	0.91	0.88, 0.94	<0.001
Model 2	0.91	0.87, 0.95	<0.001	0.96	0.93, 0.99	0.013
Model 3	0.91	0.87, 0.95	<0.001	0.96	0.93, 0.99	0.019

Model 1 did not include any covariate adjustments; Model 2 adjusted for Age, Gender, Educational attainment, Marital status, Living situation, Residence, and Economic status; Model 3 further adjusted for Smoking, Drinking, Exercise, BMI (kg/m^2^), Vitamin A, C, and E supplementation, and Chronic disease. Abbreviation: *OR*, odds ratio; *CI*, confidence interval.

**Table 5:** Mediation analysis of psychological resilience on the cMIND diet and cognitive trajectories.

Variable	Effect	Coefficient	Bootstrap 95% *CI*	*p*
cMIND diet scores	X → M	0.31	0.19, 0.43	<0.001
	M → Y	0.01	0.00, 0.02	0.085
	X → Y	−0.02	−0.04, 0.00	0.198
	a * b	0.00	−0.01, 0.00	0.096

Note: Adjusted for all covariates. X represented cMIND diet scores; M represented resilience score as a mediator; Y represented cognitive trajectory group; a * b represented indirect effect. The coefficient and 95% CI rounded to two decimals were 0.00 (−0.01, 0.00); the more precise values were a * b = −0.003 (−0.007, 0.000). Abbreviation: cMIND, the Chinese version of the Mediterranean–DASH (Dietary Approaches to Stop Hypertension) Intervention for Neurodegenerative Delay; *CI*, confidence interval.

## Data Availability

The datasets analyzed during the current study are available in the Chinese Longitudinal Healthy Longevity Survey (CLHLS) repository, [https://doi.org/10.18170/DVN/WBO7LK].

## Supplementary Materials

The following supporting information can be downloaded at https://www.mdpi.com/article/10.3390/nu18213608/s1. Table S1: Baseline characteristics of the overall, included, and excluded samples. Table S2: The cMIND diet scale. Table S3: The Chinese Mini-Mental State Examination (cMMSE) in the CLHLS. Table S4: The Simplified Resilience Scale (SRS) in the CLHLS. Table S5: Parameters in cognitive trajectory modeling. Table S6. Association between single food item in the cMIND diet and cognitive trajectories. Table S7: Association between covariates and cognitive trajectories. Table S8: Baseline characteristics of the overall sample and different trajectory groups in the first sensitivity analysis. Table S9: Association between the cMIND diet pattern and cognitive trajectories in the first sensitivity analysis. Table S10: Association between covariates and cognitive trajectories in the first sensitivity analysis. Table S11: Mediation analysis of psychological resilience on cMIND diet and cognitive trajectories in the first sensitivity analysis. Table S12: Baseline characteristics of the overall sample and different trajectory groups in the second sensitivity analysis. Table S13: Association between the cMIND diet pattern and cognitive trajectories in the second sensitivity analysis. Table S14: Association between covariates and cognitive trajectories in the second sensitivity analysis. Table S15: Association between the cMIND diet and psychological resilience in the second sensitivity analysis. Table S16: Association between psychological resilience and cognitive trajectories in the second sensitivity analysis. Table S17: Mediation analysis of psychological resilience on the cMIND diet and cognitive trajectories in the second sensitivity analysis. Figure S1: One cognitive trajectory. Figure S2: Two cognitive trajectories. Figure S3: Four cognitive trajectories. Figure S4: Five cognitive trajectories.

## Abbreviations

The following abbreviations are used in this manuscript:

cMIND	The Chinese version of the Mediterranean Dietary Approaches to Stop Hypertension Intervention for Neurodegenerative Delay
CLHLS	the Chinese Longitudinal Healthy Longevity Survey
GBTM	A group-based trajectory model
MCI	Mild cognitive impairment
cMMSE	The Chinese Mini-Mental State Examination
AvePP	Average posterior probability
OCCs	Odds of correct classification
AIC	Akaike Information Criterion
BIC	Bayesian Information Criterion
CAIC	Consistent Akaike Information Criterion
SABIC	Sample-Size Adjusted Bayesian Information Criterion
SCFAs	Short-chain fatty acids
BMI	body mass index
SRS	The Simplified Resilience Scale
*OR*	Odds ratio
*CI*	Confidence interval
Ref	Reference category
VIF	Variance Inflation Factor
