# Supplementary material for: Association Between the cMIND Diet Pattern, Psychological Resilience and Cognitive Decline Trajectories: Findings from a Prospective Cohort Study in China
Source: Nutrients. 2026 Oct 30;18(21):3608. doi: 10.3390/nu18213608 (PMC13765467; doi:10.3390/nu18213608)
Supplement: Supplementary file 1 [file nutrients-18-03608-s001.zip › nutrients-4611093-supplementary.pdf]

Appendix A

Table S1. Baseline characteristics of the overall, included, and excluded samples.

**Variable**	**Category**	**Total sample (n = 16954)**	**Groups**		**U/X^2**	***p***
			**Included (n = 1755)**	**Excluded (n = 15199)**		
Age		89.0 (78.0, 96.0)	73.0 (69.0, 80.0)	90.0 (81.0, 98.0)	−45.15	< 0.001
Gender	male	7252 (42.8)	828 (47.2)	6424 (42.3)	15.52	< 0.001
	female	9702 (57.2)	927 (52.8)	8775 (57.7)		
Educational attainment	low	10567 (62.3)	844 (48.1)	9723 (64.0)	169.35	< 0.001
	medium	4679 (27.6)	674 (38.4)	4005 (26.4)		
	high	1708 (10.1)	237 (13.5)	1471 (9.7)		
Marital status	married	5535 (32.6)	1095 (62.4)	4440 (29.2)	790.40	< 0.001
	divorced	49 (0.3)	3 (0.2)	46 (0.3)		
	widowed	11216 (66.2)	642 (36.6)	10574 (69.6)		
	single	154 (0.9)	15 (0.9)	139 (0.9)		
Living situation	with family	14084 (83.1)	1476 (84.1)	12608 (83.0)	17.06	< 0.001
	living alone	2562 (15.1)	269 (15.3)	2293 (15.1)		
	nursing home	308 (1.8)	10 (0.6)	298 (2.0)		
Residence	rural	13603 (80.2)	1525 (86.9)	12078 (79.5)	54.75	< 0.001
	urban	3351 (19.8)	230 (13.1)	3121 (20.5)		
Economic status	good	2255 (13.3)	220 (12.5)	2035 (13.4)	14.7	< 0.001
	normal	11619 (68.5)	1269 (72.3)	10350 (68.1)		
	poor	3080 (18.2)	266 (15.2)	2814 (18.5)		

https://doi.org/10.3390/nu18213608

Smoking	yes	2966 (17.5)	398 (22.7)	2568 (16.9)	36.44	< 0.001
	no	13988 (82.5)	1357 (77.3)	12631 (83.1)		
Drinking	yes	2933 (17.3)	391 (22.3)	2542 (16.7)	33.93	< 0.001
	no	14021 (82.7)	1364 (77.7)	12657 (83.3)		
Exercise	yes	4645 (27.4)	622 (35.4)	4023 (26.5)	63.65	< 0.001
	no	12308 (72.6)	1133 (64.6)	11175 (73.5)		
BMI (kg/m^2)	underweight	5567 (32.8)	366 (20.9)	5201 (34.2)	163.27	< 0.001
	normal	9893 (58.4)	1140 (65.0)	8753 (57.6)		
	overweight	1297 (7.6)	213 (12.1)	1084 (7.1)		
	obese	197 (1.2)	36 (2.1)	161 (1.1)		
Vitamin A, C, and E supplementation	yes	2200 (13.0)	191 (10.9)	2009 (13.2)	7.595	0.006
	no	14754 (87.0)	1564 (89.1)	13190 (86.8)		
Chronic disease	yes	8762 (51.7)	924 (52.6)	7838 (51.6)	0.74	0.391
	no	8192 (48.3)	831 (47.4)	7361 (48.4)		
cMIND diet score		5.0 (4.0, 6.0)	5.0 (4.0, 6.0)	5.0 (3.5, 6.0)	9.01	< 0.001
cMIND diet group	lower	6126 (36.1)	5640 (37.1)	486 (27.7)	67.08	< 0.001
	medium	6249 (36.9)	5557 (36.6)	692 (39.4)		
	higher	4579 (27.0)	4002 (26.3)	577 (32.9)		
SRS score		25.0 (22.0, 28.0)	25.0 (22.0, 28.0)	26.0 (24.0, 29.0)	13.16	< 0.001

Continuous variables were presented as $\bar{x}$ ± SD or median (IQR), categorical variables as *n* (%). Group differences for continuous variables (Age, cMIND diet score, and SRS score) were assessed using the Mann–Whitney U test; group differences for the remaining categorical variables were assessed using R×C chi-square tests. Abbreviation: BMI, body mass index; cMIND, the Chinese version of the Mediterranean–DASH (Dietary Approaches to Stop Hypertension) Intervention for

https://doi.org/10.3390/nu18213608

Neurodegenerative Delay; SRS, the Simplified Resilience Scale. The Chinese version of the Mediterranean–DASH (Dietary Approaches to Stop Hypertension) Intervention for Neurodegenerative Delay (cMIND) diet score ranges from 0 to 12; The Simplified Resilience Scale (SRS) score ranges from 5 to 35.

–
–
–

https://doi.org/10.3390/nu18213608

Table S2. The cMIND diet scale.

Components	Score		
	**0**	**0.5**	**1**
Staple food composition	Rice/wheat		Whole grains
Staple food quantity	< 250g or > 400g/day		250g–400g/day
Fresh vegetables	≤ 2 servings/week	3–5/week	≥ 6 servings/week
Mushroom or algae	≤ 1 meal/week	1–3/week	≥ 4 meals/week
Fresh fruit	≤ 2 servings/week	3–5/week	≥ 6 servings/week
Cooking oil	Animal oil		Vegetable oil
Fish	< 1/month	1–3/month	≥ 1 meal/week
Food made from beans	< 1 meal/week	1–3 meals/week	≥ 4 meals/week
Nut	< 1 serving/week	1–4/week	≥ 5 servings/week
Garlic	< 1 meal/week	1–3/week	≥ 4 meals/week
Tea	Not almost every day		almost every day
Sugar	≥ 2 servings/week	1/month–1/week	< 1 serving/month

The total cMIND diet score ranges from 0 to 12 points. Abbreviation: cMIND, the Chinese version of the Mediterranean–DASH (Dietary Approaches to Stop Hypertension) Intervention for Neurodegenerative Delay.

Table S3. The Chinese Mini-Mental State Examination (cMMSE) in the CLHLS.

Domins	Questions	Scores
Orientation (12 marks)	1. What time of day is it right now (morning, afternoon, evening)?	1
	2. What is the month (Western or Chinese calendar) right now?	1
	3. What is the date (Chinese calendar day and month) of the mid-autumn festival?	1
	4. What is the season right now, spring, summer, fall, winter?	1
	5. What is the name of this district or town?	1
	6. Please name as many kinds of food as possible in 1 minute.	7
Registration (3 marks)	1. I am now going to test your memory. I will mention three objects. Please repeat these three objects (table, apple, clothes).	3
Attention and calculation (6 marks)	1. I will ask you to spend 3 dollars from 20 dollars, then you must spend 3 dollars from the number you arrived at and continue to spend 3 dollars until you are asked to stop.	5
	2. Ask the interviewee to draw the figure on B Card.	1
Recall (3 marks)	1. Please repeat the three words (in any order) that I asked you to repeat a little while ago.	3
Language (6 marks)	1. Give the interviewee a pen and then a watch and ask what these objects are called (pen, watch).	2
	2. I will now ask you to repeat the following sentence: 'What you plant, what you will get.'	1
	3. I will give you a piece of paper. You must take the paper using your right hand, fold it in the middle using both hands, and place the paper on the floor.	3

The cMMSE score ranges from 0 to 30 points.

https://doi.org/10.3390/nu18213608

Table S4. The Simplified Resilience Scale (SRS) in the CLHLS.

Number	Questions	Responds
1	Can you manage to keep a positive outlook no matter what happens?	1. Always 2. Often 3. Sometimes 4. Rarely 5. Never
2	Do you like to keep things clean and tidy?	1. Always 2. Often 3. Sometimes 4. Rarely 5. Never
3	Do you often feel anxious or afraid?	1. Always 2. Often 3. Sometimes 4. Rarely 5. Never
4	Do you often feel lonely?	1. Always 2. Often 3. Sometimes 4. Rarely 5. Never
5	Can you make your own decisions about your affairs?	1. Always 2. Often 3. Sometimes 4. Rarely 5. Never
6	Do you feel that you are becoming less capable as you get older?	1. Always 2. Often 3. Sometimes 4. Rarely 5. Never
7	Do you feel as cheerful as you did when you were younger?	1. Always 2. Often 3. Sometimes 4. Rarely 5. Never

The SRS score ranges from 5 to 35 points.

Table S5. Parameters in cognitive trajectory modeling.

Model	AvePP	Mean_APPA	Prior	AIC	BIC	CAIC	SABIC	OCCs
Traj_1	1.00	1	100%	42650.05	42677.47	42681.47	42664.76	1
Traj_2	0.96/0.97	0.97	34.4%/65.6%	36478.13	36539.84	36548.84	36511.24	44.64/17.51
Traj_3	0.96/0.093/0.94	0.95	20.0%/30.8%/49.2%	34874.43	34970.42	34984.42	34925.93	89.26/31.50/17.68
Traj_4	0.84/0.94/0.94/0.86	0.89	11.1%/31.3%/49.4%/8.2%	34751.07	34881.34	34900.34	34820.96	41.06/34.14/15.78/66.31
Traj_5	0.91/0.92/0.90/0.86/0.87	0.89	36.2%/11.4%/37.4%/9.0%/6.0%	34200.82	34365.38	34389.38	34289.11	18.52/85.18/15.41/61.49102.19

Abbreviation: Traj_1, one cognitive trajectory; Traj_2, two cognitive trajectories; Traj_3, three cognitive trajectories; Traj_4, four cognitive trajectories; Traj_5, five cognitive trajectories; AvePP,

https://doi.org/10.3390/nu18213608

Table S6. Association between single food item in the cMIND diet and cognitive trajectories.

Single food item	Total (*n* = 1755)	Trajectory Groups			*p*	Rapid decline vs. Stable		Slow decline vs. Stable	
		Rapid-decline (*n* = 344)	Stable (*n* = 551)	Slow-decline (*n* = 860)		*OR* (95 *CI*%)	*p*	*OR* (95 *CI*%)	*p*
Consumption of whole grain									
Yes	55 (3.1)	20 (2.9)	18 (3.3)	27 (3.1)	0.956	1.10 (0.41–2.90)	0.86	1.32 (0.66–2.66)	0.43
No	1700 (96.9)	334 (97.1)	533 (96.7)	833 (96.9)		1.00 (Ref)		1.00 (Ref)	
Consumption of a suitable amount of staple food									
Yes	1007 (57.4)	196 (57.0)	301 (54.6)	510 (59.3)	0.220	0.95 (0.68–1.31)	0.734	1.13 (0.89–1.43)	0.334
No	748 (42.6)	148 (43.0)	250 (45.4)	350 (40.7)		1.00 (Ref)		1.00 (Ref)	
Consumption of fresh fruits									
Yes	1426 (81.3)	265 (77.0)	469 (85.1)	692 (80.5)	0.008	0.64 (0.42–0.97)	0.035	0.76 (0.55–1.05)	0.095
No	329 (18.7)	79 (23.0)	82 (14.9)	168 (19.5)		1.00 (Ref)		1.00 (Ref)	
Consumption of vegetables									
Yes	1737 (99.0)	338 (98.3)	548 (99.5)	851 (99.0)	0.222	0.43 (0.08–2.43)	0.336	0.532 (0.12–2.31)	0.4
No	18 (1.0)	6 (1.7)	3 (0.5)	9 (1.0)		1.00 (Ref)		1.00 (Ref)	
Consumption of plant oil									
Yes	1472 (83.9)	277 (80.5)	483 (87.7)	712 (82.8)	0.009	0.66 (0.42–1.03)	0.065	0.77 (0.54–1.08)	0.132
No	283 (16.1)	67 (19.5)	68 (12.3)	148 (16.1)		1.00 (Ref)		1.00 (Ref)	
Consumption of fish									
Yes	1107 (63.1)	205 (59.6)	380 (69.0)	522 (60.7)	0.002	0.76 (0.54–1.07)	0.114	0.80 (0.62–1.03)	0.082
No	648 (36.9)	139 (40.4)	171 (31.0)	338 (39.3)		1.00 (Ref)		1.00 (Ref)	
Consumption of bean									
Yes	1254 (71.5)	235 (68.3）	421 (76.4)	598 (69.5)	0.007	0.79 (0.55–1.13)	0.189	0.83 (0.63–1.09)	0.172

https://doi.org/10.3390/nu18213608

No	501 (28.5)	109 (31.7)	130 (23.6)	262 (30.5)		1.00 (Ref)		1.00 (Ref)	
Consumption of sugar									
Yes	758 (43.2)	150 (43.6)	218 (39.6)	390 (45.3)	0.100	0.93 (0.67–1.30)	0.674	1.18 (0.93–1.50)	0.169
No	997 (56.8)	194 (56.4)	333 (60.4)	470 (54.7)		1.00 (Ref)		1.00 (Ref)	
Consumption of tea									
Yes	639 (36.4)	112 (32.6)	232 (42.1)	295 (34.3)	0.003	0.92 (0.65–1.29)	0.613	0.85 (0.66–1.08)	0.181
No	1116 (63.6)	232 (67.4)	319 (57.9)	565 (65.7)		1.00 (Ref)		1.00 (Ref)	
Consumption of garlic									
Yes	1023 (58.3)	184 (53.5)	338 (61.3)	501 (58.3)	0.068	0.0.98 (0.70–1.36)	0.896	1.05 (0.82–1.34)	0.711
No	732 (41.7)	160 (46.5)	213 (38.7)	359 (41.7)		1.00 (Ref)		1.00 (Ref)	
Consumption of nut									
Yes	488 (27.8)	72 (20.9)	185 (33.6)	231 (26.9)	<0.001	0.96 (0.65–1.40)	0.818	1.01 (0.77–0.31)	0.971
No	1266 (72.2)	272 (79.1)	366 (66.4)	628 (73.1)		1.00 (Ref)		1.00 (Ref)	
Consumption of algae/mushrooms									
Yes	476 (27.1)	73 (21.2)	189 (34.3)	214 (24.9)	<0.001	1.01 (0.68–1.50)	0.959	0.89 (0.68–1.18)	0.417
No	1278 (72.9)	271 (78.8)	362 (65.7)	645 (75.1)		1.00 (Ref)		1.00 (Ref)	

Adjusted for all covariates. Note: For statistical analysis, the frequency responses were dichotomized: “almost every day” and “≥ once per week” were coded as “Yes consumption”; the remaining categories (“≥ once per month”, “occasionally”, and “rarely or never”) were coded as “No consumption”. Abbreviation: *OR*, odds ratio; *CI*, confidence interval; Ref, reference category.

https://doi.org/10.3390/nu18213608

Table S7. Association between covariates and cognitive trajectories.

Variable	Category	Rapid decline vs. Stable			Slow decline vs. Stable		
		OR	95% *CI*	*p*	*OR*	95% *CI*	*p*
Age	validated age	1.21	1.17, 1.24	< 0.001	1.09	1.07, 1.12	< 0.001
Gender	male	0.42	0.27, 0.64	< 0.001	0.61	0.45, 0.83	0.001
	female	1.00 (Ref)			1.00 (Ref)		
Educational attainment	low	12.33	5.42, 28.04	< 0.001	3.85	2.62, 5.65	< 0.001
	medium	3.71	1.65, 8.35	0.002	1.67	1.19, 2.35	0.003
	high	1.00 (Ref)			1.00 (Ref)		
Marital status	married	1.14	0.17, 7.86	0.895	0.93	0.26, 3.37	0.910
	Divorced [a]	—	—	—	—	—	—
	widowed	1.27	0.19, 8.65	0.810	0.89	0.25, 3.18	0.852
	single	1.00 (Ref)			1.00 (Ref)		
Living situation	with family	2.40	0.30, 19.60	0.413	2.35	0.51, 10.85	0.273
	living alone	3.33	0.40, 27.73	0.266	3.34	0.71, 15.70	0.126
	nursing home	1.00 (Ref)			1.00 (Ref)		
Residence	rural	1.28	0.73, 2.22	0.388	1.10	0.75, 1.61	0.613
	urban	1.00 (Ref)			1.00 (Ref)		
Economic status	good	0.60	0.32, 1.13	0.114	0.62	0.39, 1.00	0.049
	normal	0.76	0.48, 1.20	0.242	0.86	0.60, 1.22	0.391
	poor	1.00 (Ref)			1.00 (Ref)		
Smoke	yes	0.99	0.62, 1.56	0.948	0.98	0.72, 1.32	0.871
	no	1.00 (Ref)			1.00 (Ref)		
Drink	yes	1.14	0.74, 1.77	0.559	1.06	0.79, 1.44	0.693
	no	1.00 (Ref)			1.00 (Ref)		
Exercise	yes	0.83	0.57, 1.20	0.315	1.05	0.81, 1.37	0.696
	no	1.00 (Ref)			1.00 (Ref)		
BMI (kg/m^2)	underweight	0.40	0.13, 1.24	0.112	1.19	0.47, 2.96	0.716
	normal	0.31	0.11, 0.91	0.034	0.99	0.41, 2.38	0.985
	overweight	0.38	0.12, 1.18	0.094	0.76	0.31, 1.91	0.562
	obese	1.00 (Ref)			1.00 (Ref)		
Vitamin A, C, and E supplementation	yes	0.79	0.46, 1.35	0.386	0.81	0.55, 1.19	0.285
	no	1.00 (Ref)			1.00 (Ref)		
Chronic disease	yes	0.96	0.69, 1.34	0.802	1.16	0.91, 1.47	0.245

	no	1.00 (Ref)	1.00 (Ref)

Adjusted for all covariates. Abbreviation: BMI, body mass index; *OR*, odds ratio; *CI*, confidence interval; Ref, reference category. [a] The "divorced" category contained very few cases (n = 3, 0.2% of the total sample), the corresponding model outputs — including the *OR* (e.g., *OR*: 7.669×10^{-8}), 95% *CI*, and P value — yielded implausibly small estimates that deviated markedly from the expected distribution, therefore the effect estimate for the divorced group was considered unreliable and was not reported.

Table S8. Baseline characteristics of the overall sample and different trajectory groups in the first sensitivity analysis.

Variable	Category	Total sample (n = 4184)	Trajectory Groups			H/X^2	p
			Rapid-decline (n = 1036)	Stable (n = 1258)	Slow-decline (n = 1890)		
Age		77.0 (70.0, 84.0)	85.0 (79.0, 91.0)	72.0 (68.0, 77.0)	76.0 (71.0, 83.0)	931.87	< 0.001
Gender	male	2015 (48.2)	325 (31.4)	810 (64.4)	880 (46.6)	251.60	< 0.001
	female	2169 (51.8)	711 (68.6)	448 (35.6)	1010 (53.4)		
Educational attainment	low	2142 (51.2)	777 (75.0)	331 (26.3)	1034 (54.7)	680.37	< 0.001
	medium	1505 (36.0)	218 (21.0)	582 (46.3)	705 (37.3)		
	high	537 (12.8)	41 (4.0)	345 (27.4)	151 (8.0)		
Marital status	married	2299 (55.0)	331 (31.9)	907 (72.1)	1061 (56.1)		< 0.001
	divorced	9 (0.2)	2 (0.2)	6 (0.5)	1 (0.0)		
	widowed	1842 (44.0)	698 (67.4)	334 (26.6)	810 (42.9)		
	single	34 (0.8)	5 (0.5)	11 (0.9)	18 (1.0)		
Living situation	with family	3483 (83.3)	823 (79.4)	1111 (88.3)	1549 (82.0)	37.17	< 0.001
	living alone	669 (16.0)	205 (19.8)	138 (11.0)	326 (17.2)		
	nursing home	32 (0.7)	8 (0.8)	9 (0.7)	15 (0.8)		
Residence	rural	3535 (84.5)	896 (86.5)	981 (78.0)	1658 (87.7)	58.91	< 0.001
	urban	649 (15.5)	140 (13.5)	277 (22.0)	232 (12.3)		
Economic status	good	593 (14.2)	138 (13.3)	236 (18.7)	219 (11.6)	66.60	< 0.001
	normal	2926 (69.9)	683 (65.9)	884 (70.3)	1359 (71.9)		
	poor	665 (15.9)	215 (20.8)	138 (11.0)	312 (16.5)		
Smoking	yes	977 (23.4)	175 (16.9)	382 (30.4)	420 (22.2)	60.08	< 0.001
	no	3207 (76.6)	861 (83.1)	876 (69.6)	1470 (77.8)		
Drinking	yes	892 (21.3)	159 (15.3)	333 (26.5)	400 (21.2)	41.95	< 0.001
	no	3292 (78.7)	877 (84.7)	925 (73.5)	1490 (78.8)		

https://doi.org/10.3390/nu18213608

Exercise	yes	1543 (36.9)	299 (28.9)	570 (45.3)	674 (35.7)	68.23	< 0.001
	no	2641 (63.1)	737 (71.1)	688 (54.7)	1216 (64.3)		
BMI (kg/m^2)	underweight	958 (22.9)	330 (31.9)	183 (14.6)	445 (23.6)	126.07	< 0.001
	normal	2699 (64.5)	618 (59.7)	852 (67.7)	1229 (65.0)		
	overweight	458 (10.9)	71 (6.8)	198 (15.7)	189 (10.0)		
	obesity	69 (1.7)	17 (1.6)	25 (2.0)	27(1.4)		
Vitamin supplementation	yes	500 (12.0)	117 (11.3)	191 (15.2)	192 (10.2)	18.68	< 0.001
	no	3684 (88.0)	919 (88.7)	1067 (84.8)	1698 (89.8)		
Chronic disease	yes	2321 (55.5)	528 (51.0)	695 (55.3)	1098 (58.1)	13.81	0.001
	no	1863 (44.5)	508 (49.0)	563 (44.7)	792 (41.9)		
cMIND diet score		5.5 (4.5, 6.5)	5.0 (4.0, 6.0)	6.0 (4.5, 7.0)	5.0 (4.0, 6.5)	138.85	< 0.001
cMIND diet group	lower	1023 (24.5)	319 (30.8)	213 (17.0)	491 (26.0)	119.63	< 0.001
	medium	1535 (36.7)	404 (39.00)	414 (32.9)	717 (37.9)		
	higher	1625 (38.8)	313 (30.2)	630 (50.1)	682 (36.1)		
Resilience score		26.0 (24.0, 29.0)	25.0 (22.0, 28.0)	27.0 (25.0, 30.0)	26.0 (24.0, 29.0)	191.49	< 0.001

Continuous variables were presented as $\bar{x}$ ± SD or median (IQR), categorical variables as *n* (%). Group differences for continuous variables (Age, cMIND diet score, and SRS score) were assessed using the Kruskal–Wallis H test; group differences for the remaining categorical variables were assessed using R×C chi-square tests. Abbreviation: BMI, body mass index; cMIND, the Chinese version of the Mediterranean–DASH (Dietary Approaches to Stop Hypertension) Intervention for Neurodegenerative Delay; SRS, the Simplified Resilience Scale. The Chinese version of the Mediterranean–DASH (Dietary Approaches to Stop Hypertension) Intervention for Neurodegenerative Delay (cMIND) diet score ranges from 0 to 12; the Simplified Resilience Scale (SRS) score ranges from 5 to 35.

https://doi.org/10.3390/nu18213608

Table S9. Association between the cMIND diet pattern and cognitive trajectories in the first sensitivity analysis.

Model	Diet group	Rapid-decline vs. Stable			Slow-decline vs. Stable		
		OR	95% *CI*	*p*	*OR*	95% *CI*	*p*
Model 1	lower	3.01	2.42, 3.76	< 0.001	2.13	1.75, 2.59	< 0.001
	medium	1.96	1.25, 2.40	< 0.001	1.60	1.36, 1.88	< 0.001
	higher	1.00 (Ref)			1.00 (Ref)		
Model 2	lower	1.61	1.22, 2.11	< 0.001	1.51	1.21, 1.87	< 0.001
	medium	1.27	1.01, 1.60	0.046	1.21	1.01, 1.45	0.035
	higher	1.00 (Ref)			1.00 (Ref)		
Model 3	lower	1.53	1.16, 2.02	0.002	1.44	1.15, 1.79	0.001
	medium	1.24	0.98, 1.57	0.073	1.19	0.99, 1.42	0.060
	higher	1.00 (Ref)			1.00 (Ref)		

Model 1 did not include any covariate adjustments; Model 2 adjusted for Age, Gender, Educational attainment, Marital status, Living situation, Residence, and Economic status; Model 3 further adjusted for Smoking, Drinking, Exercise, BMI (kg/m^2), Vitamin A, C, and E supplementation, and Chronic disease. Abbreviation: cMIND, the Chinese version of the Mediterranean–DASH (Dietary Approaches to Stop Hypertension) Intervention for Neurodegenerative Delay; *OR*, odds ratio; *CI*, confidence interval; Ref, reference category.

Table S10. Association between covariates and cognitive trajectories in the first sensitivity analysis.

Variable	Category	Rapid-decline vs. Stable			Slow-decline vs. Stable		
		OR	*95% CI*	*p*	*OR*	*95% CI*	*p*
Age	validated age	1.17	1.15, 1.18	< 0.001	1.07	1.05, 1.08	< 0.001
Gender	male	0.42	0.33, 0.55	< 0.001	0.61	0.51, 0.76	< 0.001
	female	1.00 (Ref)			1.00 (Ref)		
Educational attainment	low	6.41	4.26, 9.65	< 0.001	3.85	2.97, 4.99	< 0.001
	medium	2.35	1.57, 3.51	< 0.001	2.17	1.72, 2.75	< 0.001
	high	1.00 (Ref)			1.00 (Ref)		
Marital status	married	1.58	0.43, 5.76	0.487	1.07	0.47, 2.46	0.867
	divorced	5.26	0.55, 50.63	0.150	0.23	0.02, 2.33	0.212
	widowed	2.23	0.62, 8.08	0.222	1.11	0.49, 2.53	0.805
	single	1.00 (Ref)			1.00 (Ref)		
Living situation	with family	1.44	0.47, 4.44	0.522	0.92	0.38, 2.23	0.850
	living alone	1.43	0.46, 4.47	0.540	1.11	0.45, 2.74	0.814
	nursing home	1.00 (Ref)			1.00 (Ref)		
Residence	rural	1.16	0.86, 1.58	0.337	1.33	1.05, 1.68	0.020
	urban	1.00 (Ref)			1.00 (Ref)		
Economic status	good	0.47	0.32, 0.68	< 0.001	0.60	0.44, 0.81	< 0.001
	normal	0.65	0.49, 0.86	0.003	0.86	0.68, 1.09	0.201
	poor	1.00 (Ref)			1.00 (Ref)		
Smoke	yes	1.13	0.86, 1.49	0.381	0.94	0.77, 1.14	0.525
	no	1.00 (Ref)			1.00 (Ref)		
Drink	yes	0.94	0.72, 1.24	0.678	1.05	0.86, 1.29	0.615
	no	1.00 (Ref)			1.00 (Ref)		
Exercise	yes	0.80	0.64, 1.00	0.050	0.94	0.79, 1.11	0.467
	no	1.00 (Ref)			1.00 (Ref)		
BMI	underweight	0.87	0.39, 1.93	0.728	1.26	0.67, 2.38	0.469
	normal	0.67	0.31, 1.46	0.309	1.02	0.56, 1.88	0.949
	overweight	0.56	0.24, 1.28	0.170	0.83	0.44, 1.57	0.569
	obese	1.00 (Ref)			1.00 (Ref)		
Vitamin supplementation	yes	1.22	0.88, 1.67	0.386	1.26	0.98, 1.61	0.067
	no	1.00 (Ref)			1.00 (Ref)		

https://doi.org/10.3390/nu18213608

Chronic disease	yes	0.88	0.71, 1.07	0.199	0.74	0.63, 0.87	< 0.001
	no	1.00 (Ref)			1.00 (Ref)		
cMIND diet group	low	1.53	1.16, 2.02	0.002	1.44	1.15, 1.79	< 0.01
	medium	1.24	0.98, 1.57	0.073	1.19	0.99, 1.42	0.060
	high	1.00 (Ref)			1.00 (Ref)		

Adjusted for all covariates. Abbreviation: BMI, body mass index; *OR*, odds ratio; *CI*, confidence interval; Ref, reference category.

Table S11. Mediation analysis of psychological resilience on the cMIND diet and cognitive trajectories in the first sensitivity analysis.

Variable	Effect	Coefficient	Bootstrap 95% *CI*	*P*
cMIND diet scores	X→M	0.26	0.18, 0.33	< 0.001
	M→Y	0.01	0.00, 0.01	0.052
	X→Y	0.02	0.01, 0.04	0.009
	a*b	0.00	0.00, 0.00	0.065

Adjusted for all covariates. X represented cMIND diet scores; M represented resilience score as a mediator; Y represented cognitive trajectory group; a*b represented indirect effect. The coefficient and 95% *CI* rounded to two decimals were 0.00 (0.00, 0.00); the more precise values were a*b = 0.002 (0.000, 0.0004). Abbreviation: cMIND, the Chinese version of the Mediterranean–DASH (Dietary Approaches to Stop Hypertension) Intervention for Neurodegenerative Delay; CI, confidence interval.

Table S12. Baseline characteristics of the overall sample and different trajectory groups in the second sensitivity analysis.

**Variable**	**Category**	**Total sample (*n* = 1755)**	**Trajectory Groups**			*H*/X^2	*p*
			**Rapid-decline (*n* = 344)**	**Stable (*n* = 551)**	**Slow-decline (*n* = 860)**		
Age		73.0 (69.0, 80.0)	80.5 ± 7.8	70.0 (67.0, 74.0)	73.0 (69.0, 79.0)	328.22	<0.001
Gender	male	828 (47.2)	105 (30.5)	345 (62.6)	378 (44.0)	94.56	<0.01
	female	927 (52.8)	239 (69.5)	206 (37.4)	482 (56.0)		
Educational attainment	low	844 (48.1)	253 (73.5)	127 (23.0)	464 (54.0)	282.03	<0.01
	medium	674 (38.4)	83 (24.1)	274 (49.7)	317 (36.9)		
	high	237 (13.5)	8 (2.3)	150 (27.2)	79 (9.2)		
Marital status	married	1095 (62.4)	147 (42.7)	419 (76.0)	529 (61.5)		<0.01
	divorced	3 (0.2)	0 (0.0)	3 (0.5)	0 (0.0)		
	widowed	642 (36.6)	195 (56.7)	125 (22.7)	322 (37.4)		
	single	15 (0.9)	2 (0.6)	4 (0.7)	9 (1.0)		
Living situation	with family	1476 (84.1)	267 (77.6)	496 (90.0)	713 (82.9)		<0.01
	living alone	269 (15.3)	75 (21.8)	51 (9.3)	143 (16.6)		
	nursing home	10 (0.6)	2 (0.6)	4 (0.7)	4 (0.5)		
Residence	rural	1525 (86.9)	311 (90.4)	454 (82.4)	760 (88.4)	15.17	<0.01
	urban	230 (13.1)	33 (9.6)	97 (17.6)	100 (11.6)		
Economic status	good	220 (12.5)	38 (11.0)	94 (17.1)	88 (10.2)	24.29	<0.01
	normal	1269 (72.3)	241 (70.1)	397 (72.1)	631 (73.4)		
	poor	266 (15.2)	65 (18.9)	60 (10.9)	141 (16.4)		
Smoking	yes	398 (22.7)	52 (15.1)	163 (29.6)	183 (21.3)	27.16	<0.01

https://doi.org/10.3390/nu18213608

	no	1357 (77.3)	292 (84.9)	388 (70.4)	677 (78.7)		
Drinking	yes	391 (22.3)	58 (16.9)	152 (27.6)	181 (21.0)	15.55	<0.01
	no	1364 (77.7)	286 (83.1)	399 (72.4)	679 (79.0)		
Exercise	yes	622 (35.4)	97 (28.2)	220 (39.9)	305 (35.5)	12.74	0.002
	no	1133 (64.6)	247 (71.8)	331 (60.1)	555 (64.5)		
BMI (kg/m^2)	underweight	366 (20.9)	102 (29.7)	75 (13.6)	189 (22.0)	45.46	<0.001
	normal	1140 (65.0)	197 (57.3)	375 (68.1)	568 (66.0)		
	overweight	213 (12.1)	34 (9.9)	89 (16.2)	90 (10.5)		
	Obesity	36 (2.1)	11 (3.2)	12 (2.2)	13 (1.5)		
Vitamin A, C, and E supplementation	yes	191 (10.9)	35 (10.2)	71 (12.9)	85 (9.9)	3.34	0.188
	no	1564 (89.1)	309 (89.8)	480 (87.1)	775 (90.1)		
Cardiometabolic and neurodegenerative diseases	yes	524 (29.9)	99 (28.8)	162 (29.4)	263 (30.6)	0.461	0.794
	no	1231 (70.1)	245 (71.2)	389 (70.6)	597 (69.4)		
Other Chronic disease	yes	623 (35.5)	122 (35.5)	193 (35.0)	308 (35.8)	0.091	0.956
	no	1132 (64.5)	222 (64.5)	358 (65.0)	552 (64.2)		
cMIND diet score		5.0 (4.0, 6.0)	5.0 (4.0, 6.0)	5.5 (4.5, 6.5)	5.0 (4.0, 6.0)	49.28	<0.001
cMIND diet group	lower	486 (27.7)	116 (33.7)	110 (20.0)	260 (30.2)	42.38	<0.01
	medium	692 (39.4)	138 (40.1)	207 (37.6)	347 (40.3)		
	higher	577 (32.9)	90 (26.2)	234 (42.5)	253 (29.4)		
SRS score		26.0 (24.0, 29.0)	25.0 (23.0, 28.0)	28.0 (25.0, 30.0)	26.0 (24.0, 29.0)	87.63	<0.001

Continuous variables were presented as $\bar{x}$ ± SD or median (IQR), categorical variables as *n* (%). Group differences for continuous variables (Age, cMIND diet score, and SRS score) were assessed using the Kruskal–Wallis H test; group differences for the remaining categorical variables were

https://doi.org/10.3390/nu18213608

assessed using R×C chi-square tests. Abbreviation: BMI, body mass index; cMIND, the Chinese version of the Mediterranean–DASH (Dietary Approaches to Stop Hypertension) Intervention for Neurodegenerative Delay; SRS, the Simplified Resilience Scale. The Chinese version of the Mediterranean–DASH (Dietary Approaches to Stop Hypertension) Intervention for Neurodegenerative Delay (cMIND) diet score ranges from 0 to 12. The Simplified Resilience Scale (SRS) score ranges from 5 to 35.

Table S13. Association between the cMIND diet pattern and cognitive trajectories in the second sensitivity analysis.

https://doi.org/10.3390/nu18213608

Model	Diet group	Rapid-decline vs. Stable			Slow-decline vs. Stable		
		OR	95% *CI*	*p*	*OR*	95% *CI*	*p*
Model 1	lower	2.74	1.92, 3.92	< 0.001	2.19	1.64, 2.91	< 0.001
	medium	1.73	1.25, 2.40	< 0.001	1.55	1.21, 1.99	< 0.001
	higher	1.00 (Ref)			1.00 (Ref)		
Model 2	lower	1.63	1.05, 2.52	0.028	1.62	1.17, 2.24	0.003
	medium	1.18	0.79, 1.74	0.421	1.23	0.94, 1.63	0.138
	higher	1.00 (Ref)			1.00 (Ref)		
Model 3	lower	1.60	1.03, 2.49	0.037	1.63	1.17, 2.25	0.004
	medium	1.16	0.78, 1.73	0.457	1.23	0.93, 1.63	0.146
	higher	1.00 (Ref)			1.00 (Ref)		

Model 1 did not include any covariate adjustments; Model 2 adjusted for Age, Gender, Educational attainment, Marital status, Living situation, Residence, and Economic status; Model 3 further adjusted for Smoking, Drinking, Exercise, BMI (kg/m^2), Vitamin A, C, and E supplementation, and Chronic disease. Abbreviation: cMIND, the Chinese version of the Mediterranean–DASH (Dietary Approaches to Stop Hypertension) Intervention for Neurodegenerative Delay; *OR*, odds ratio; *CI*, confidence interval; Ref, reference category.

Table S14. Association between covariates and cognitive trajectories in the second sensitivity analysis.

https://doi.org/10.3390/nu18213608

Variable	Category	Rapid-decline vs. Stable			Slow-decline vs. Stable		
		OR	95% *CI*	*p*	*OR*	95% *CI*	*p*
Age	validated age	1.21	1.18, 1.24	< 0.001	1.09	1.07, 1.12	< 0.001
Gender	male	0.43	0.28, 0.65	< 0.001	0.62	0.46, 0.83	0.002
	female	1.00 (Ref)			1.00 (Ref)		
Educational attainment	low	12.44	5.47, 28.29	< 0.001	3.85	2.62, 5.65	< 0.001
	medium	3.71	1.65, 8.34	0.002	1.68	1.19, 2.36	0.003
	high	1.00 (Ref)			1.00 (Ref)		
Marital status	married	1.17	0.17, 8.01	0.876	0.95	0.26, 3.45	0.943
	divorced	—	—	—	—	—	—
	widowed	1.30	0.19, 8.85	0.788	0.91	0.26, 3.27	0.888
	single	1.00 (Ref)			1.00 (Ref)		
Living situation	with family	2.42	0.30, 19.41	0.406	2.33	0.5, 10.81	0.280
	living alone	3.31	0.40, 27.19	0.265	3.33	0.7, 15.72	0.129
	nursing home	1.00 (Ref)			1.00 (Ref)		
Residence	rural	1.33	0.76, 2.32	0.316	1.13	0.77, 1.66	0.518
	urban	1.00 (Ref)			1.00 (Ref)		
Economic status	good	0.60	0.31, 1.13	0.112	0.60	0.38, 0.97	0.038
	normal	0.76	0.48, 1.21	0.246	0.83	0.58, 1.2	0.322
	poor	1.00 (Ref)			1.00 (Ref)		
Smoke	yes	0.99	0.62, 1.56	0.951	0.97	0.72, 1.32	0.856
	no	1.00 (Ref)			1.00 (Ref)		
Drink	yes	1.15	0.74, 1.79	0.526	1.08	0.8, 1.46	0.630
	no	1.00 (Ref)			1.00 (Ref)		
Exercise	yes	0.81	0.56, 1.16	0.250	1.05	0.81, 1.37	0.708
	no	1.00 (Ref)			1.00 (Ref)		
BMI	underweight	0.44	0.14, 1.34	0.146	1.22	0.49, 3.04	0.676
	normal	0.33	0.11, 0.98	0.046	1.01	0.42, 2.41	0.990
	overweight	0.39	0.12, 1.22	0.106	0.76	0.3, 1.9	0.558
	obese	1.00 (Ref)			1.00 (Ref)		
Vitamin supplementation	yes	1.30	0.76, 2.21	0.344	1.24	0.84, 1.83	0.270
	no	1.00 (Ref)			1.00 (Ref)		
Cardiometabolic and neurodegenerative diseases	yes	0.81	0.56, 1.18	0.274	0.80	0.61, 1.04	0.099
	no	1.00 (Ref)			1.00 (Ref)		
Other Chronic disease	yes	0.97	0.69, 1.36	0.855	1.01	0.79, 1.3	0.930
	no	1.00 (Ref)			1.00 (Ref)		
cMIND diet group	low	1.60	1.03, 2.49	0.037	1.63	1.17, 2.25	0.004
	medium	1.16	0.78, 1.73	0.457	1.23	0.93, 1.63	0.146
	high	1.00 (Ref)			3.85	1.07, 1.12	

Adjusted for all covariates. Abbreviation: BMI, body mass index; *OR*, odds ratio; *CI*, confidence interval; Ref, reference category.

https://doi.org/10.3390/nu18213608

Table S15. Association between the cMIND diet and psychological resilience in the second sensitivity analysis

**Model**	**Unstandardised coefficient *β***	**Standardisation coefficient *β***	**t**	**95% *CI***	***p***	**Statistics on collinearity**	
						**tolerance**	**VIF**
Model 1	0.58	0.23	10.01	0.46, 0.69	< 0.001	1.00	1.00
Model 2	0.35	0.14	6.03	0.24, 0.47	< 0.001	0.86	1.16
Model 3	0.31	0.13	5.27	0.20, 0.43	< 0.001	0.83	1.21

Model 1 did not include any covariate adjustments; Model 2 adjusted for Age, Gender, Educational attainment, Marital status, Living situation, Residence, and Economic status; Model 3 further adjusted for Smoking, Drinking, Exercise, BMI (kg/m^2), Vitamin A, C, and E supplementation, and Chronic disease.

https://doi.org/10.3390/nu18213608

Table S16. Association between psychological resilience and cognitive trajectories in the second sensitivity analysis.

**Model**	**Rapid decline vs. Stable**			**Slow decline vs. Stable**		
	OR	**95% *CI***	***p***	***OR***	**95% *CI***	***p***
Model 1	0.84	0.80, 0.87	< 0.001	0.91	0.88, 0.94	< 0.001
Model 2	0.91	0.87, 0.95	< 0.001	0.96	0.93, 0.99	0.013
Model 3	0.91	0.87, 0.96	< 0.001	0.96	0.93, 0.99	0.020

Model 1 did not include any covariate adjustments; Model 2 adjusted for Age, Gender, Educational attainment, Marital status, Living situation, Residence, and Economic status; Model 3 further adjusted for Smoking, Drinking, Exercise, BMI (kg/m^2), Vitamin A, C, and E supplementation, and Chronic disease. Abbreviation: *OR*, odds ratio; *CI*, confidence interval.

https://doi.org/10.3390/nu18213608

Table S17. Mediation analysis of psychological resilience on the cMIND diet and cognitive trajectories in the second sensitivity analysis.

Variable	Effect	Coefficient	Bootstrap 95% *CI*	*P*
cMIND diet scores	X→M	0.31	0.20, 0.43	< 0.001
	M→Y	0.01	0.00, 0.02	0.098
	X→Y	−0.02	−0.04, 0.01	0.182
	a*b	0.00	0.00, 0.01	0.120

Adjusted for all covariates. X represented cMIND diet scores; M represented resilience score as a mediator; Y represented cognitive trajectory group; a*b represented indirect effect. The coefficient and 95% *CI* rounded to two decimals were 0.00 (0.00, 0.01); the more precise values were a*b = 0.003 (−0.001, 0.0007). Abbreviation: cMIND, the Chinese version of the Mediterranean–DASH (Dietary Approaches to Stop Hypertension) Intervention for Neurodegenerative Delay; CI, confidence interval.

https://doi.org/10.3390/nu18213608

Appendix B

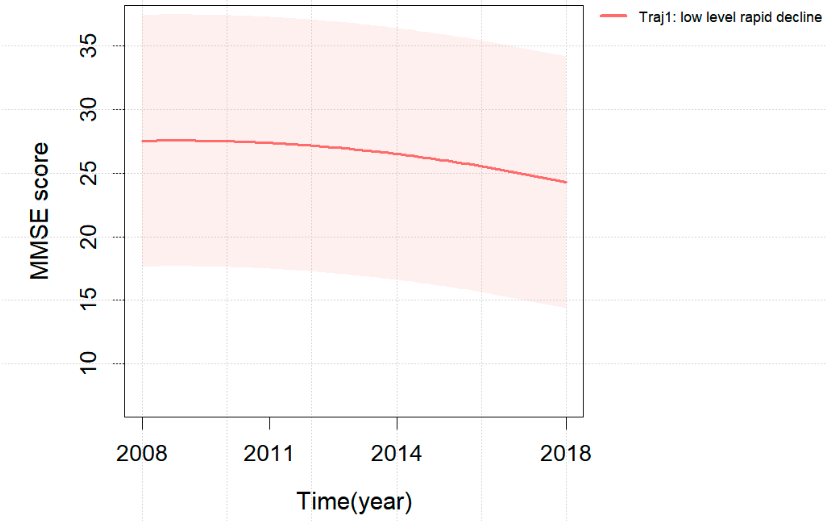

Figure S1. One cognitive trajectory.

The figure shows the decline in cMMSE score between 2008 and 2018. One cognitive decline trajectory was defined as the low-level rapid decline group. The cMMSE score ranges from 0 to 30 points. cMMSE, The Chinese Mini-Mental State Examination.

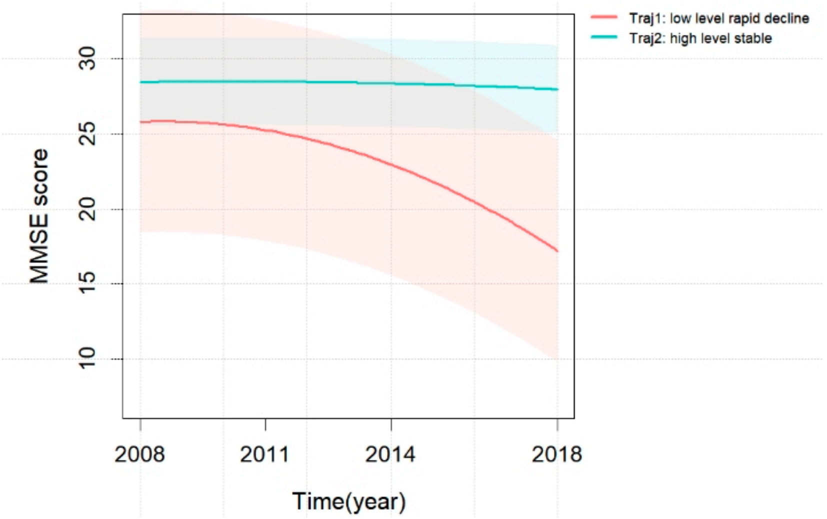

Figure S2. Two cognitive trajectories.

The figure shows the decline in cMMSE score between 2008 and 2018. Two distinct cognitive decline trajectories were defined: the low-level rapid decline group and the high-level stable group. The cMMSE score ranges from 0 to 30 points. cMMSE, The Chinese Mini-Mental State Examination.

https://doi.org/10.3390/nu18213608

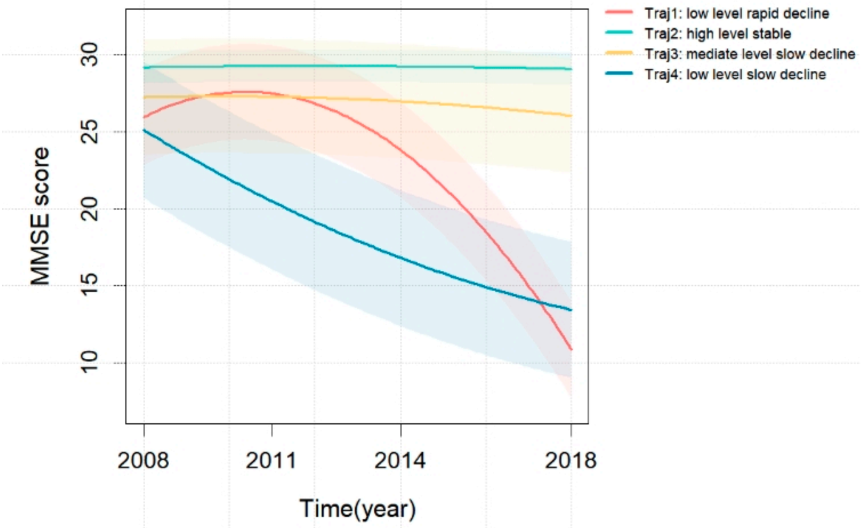

Figure S3. Four cognitive trajectories.

The figure shows the decline in cMMSE score between 2008 and 2018. Four distinct cognitive decline trajectories were defined: the low-level rapid decline group, the moderate-level slow decline group, the high-level stable group, and the low-level slow decline group. The cMMSE score ranges from 0 to 30 points. cMMSE, The Chinese Mini-Mental State Examination.

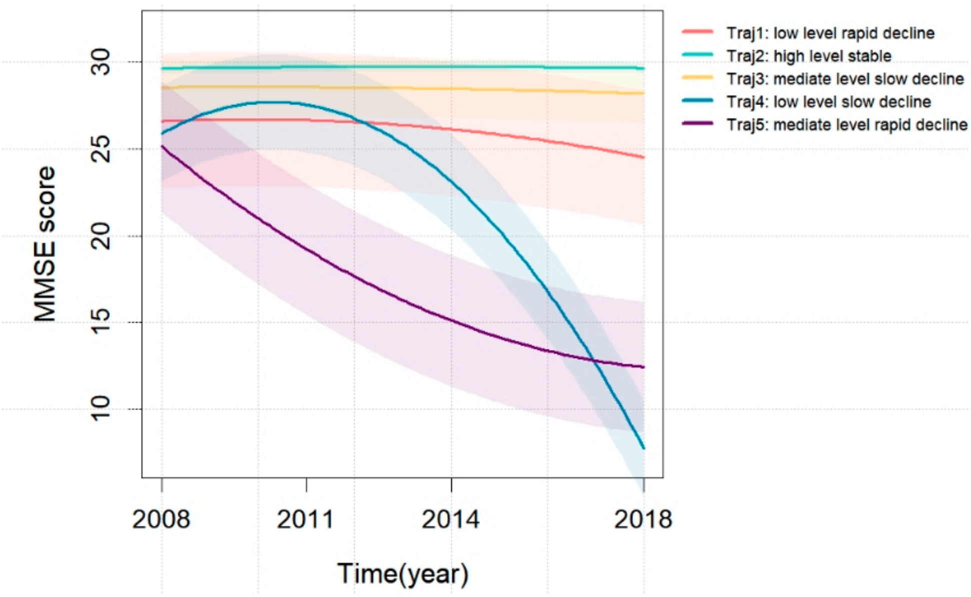

Figure S4. Five cognitive trajectories.

The figure shows the decline in cMMSE score between 2008 and 2018. Five distinct cognitive decline trajectories were defined: the low-level rapid decline group, the moderate-level slow decline group, the high-level stable group, the low-level slow decline group, and the moderate-level rapid decline group. The cMMSE score ranges from 0 to 30 points. cMMSE, The Chinese Mini-Mental State Examination.

Disclaimer/Publisher's Note: The statements, opinions and data contained in all publications are solely those of the individual author(s) and contributor(s) and not of MDPI and/or the editor(s). MDPI and/or the editor(s) disclaim responsibility for any injury to people or property resulting from any ideas, methods, instructions or products referred to in the content.

https://doi.org/10.3390/nu18213608
